# China-UK partnership for global health: practices and implications of the Global Health Support Programme 2012–2019

**DOI:** 10.1186/s41256-020-00134-7

**Published:** 2020-03-20

**Authors:** Xiaohua Wang, Peilong Liu, Tongwu Xu, Yan Chen, Yang Yu, Xun Chen, Jingyi Chen, Zhaoyang Zhang

**Affiliations:** 1 Center for Project Supervision and Management, National Health Commission of the People’s Republic of China, Beijing, 100044 China; 2 School of Public Health, Peking University, Beijing, 100191 China; 3 Graduate School of the Chinese Academy of Social Sciences, Beijing, 102488 China; 4 School of Health Sciences/Global Health Institute, Wuhan University, Wuhan, 430071 China

**Keywords:** Global health policy, Global health governance, Partnership, China, UK, Health project management

## Abstract

**Background:**

Over the past few decades, a series of major challenges to global health have successively emerged, which call for China’s deeper engagement in global health governance. In this context, the China-UK Global Health Support Programme (GHSP) was launched in 2012 with about 12 million pounds funded by the United Kingdom.

**Objectives:**

The GHSP was expected to explore a new type of China-UK partnership to strengthen the cooperation in global health, and enhance China’s capacity to engage in global health governance and provide effective development assistance in health (DAH), in order to jointly improve global health outcomes.

**Programme design and implementation:**

The GHSP was programmed to support capacity building activities in Chinese experience distillation, DAH, global health governance and pilot partnership at national and institutional levels between October 2012 and March 2019. These activities were assigned to different project implementing agencies (PIAs) and their project cooperative agencies (PCAs) or pilot areas, and were then implemented under the guidance and management by the strategic oversight committee and the project management office of GHSP respectively.

**Main achievements:**

At the national level, the GHSP held five rounds of China-UK high-level dialogues, conducted studies on China Global Health Strategies to provide robust evidence for developing and issuing relevant national policies, and supported the establishment of the China Global Health Network. At the institutional level, the GHSP funded a series of activities in research, training, international exchange and pilots etc., produced a large number of high-quality research outputs and policy briefings, cultivated a group of PIAs and individual researchers, facilitated the partnership building between the PIAs and PCAs, enhanced the practical ability of Chinese institutions to conduct overseas DAH, and improved the health service delivery and outcomes in pilot areas of three Asian and African countries.

**Policy implications:**

In the GHSP, China and UK have established a good model for North-South Cooperation and the programme facilitated the 2030 Agenda for Sustainable Development by building a new type of bilateral partnership and carrying out triangular cooperation practices. This model has demonstrated huge potential for cooperation through partnership and can also be referred to by other countries to develop bilateral partnerships.

## Background

The importance and urgency of health-related issues in global governance have never been so evident as what they are now [1]. Globalization, together with the deterioration in the environment and climate change, have led to a series of major challenges to global health development and global health security [2, 3].

In the Millennium Development Goals (MDGs), some key health indicators have yet to achieve the expected goals in 2011–2015 [4]. Inequity in global health development has become a common issue, especially in sub-Saharan Africa [5]. Non-communicable diseases (NCDs) have gradually become the main risks to human health [6]. Major public health events, especially epidemic-prone diseases with cross-border impacts, have increasingly occurred [7–11]. Other public health issues, such as anti-microbial resistance (AMR), chemical and biological terrorism, possible terrorist attacks by radiological and nuclear means, and extreme climate events, have also posed increasingly serious threats to global health security and triggered global health crises [12–14].

In addition, global development assistance for health (DAH) is in transition. In order to meet the current needs of recipient countries and improve the effectiveness, efficiency, transparency and sustainability of aid, the international aid system formed after the World War Two urgently needs reforming [15]. These global challenges cannot be addressed by one individual country. They demand joint attention, collaboration and contribution from all the stakeholders in the international community, where China is a key player.

China is a beneficiary of global health development cooperation. Since the establishment of the People’s Republic of China in 1949, it has made great efforts to improve its people’s health with only limited financial, material and medical resources [16, 17]. With the reform and opening up implemented since 1978, China has received a large amount of financial and technical assistance from international organizations and developed countries. Those supports, combined with China’s own constant dedication, have dramatically boosted the health development in China. When the China-UK Global Health Support Programme (GHSP) was set up in 2011, China had already successfully achieved Goals 4 and 5 of the MDGs, and was working hard to achieve Goal 6.

China has been a contributor to global health development cooperation. China’s population accounts for approximately one-fifth of the whole world, thus the health improvement of Chinese people is in itself a significant contribution to global health. In the past few decades, China has faithfully executed the global development agenda, vigorously supporting the work of international organizations such as the World Health Organization (WHO), and actively carrying out aid and “South-South cooperation” to help other developing countries to improve their local health status as much as possible [18–21].

China has a large potential to make greater contributions to global health. The rich experience accumulated during China’s improvement of its own health outcomes over the past 70 years has made it possible for China to provide effective public goods for global development. In recent times, the rapid growth of China’s overall national strength have made it possible for the country to turn its eyes more to the rest of the world. The Chinese government has also made commitments to global health on many major diplomatic occasions. Therefore, both the international community and China’s own health workers are expecting China to contribute further to global health.

However, compared with developed countries, there is still a substantial gap between China’s “strong willingness” and its actual “qualified capacity” to engage in global health. China is not good at distilling and disseminating Chinese experience and lessons in health with a view to external applicability. There is a lack of understanding of the best practices of international DAH, and an inadequate ability to engage in global health governance and policy making and for cross-border public health interventions and joint action. These insufficiencies have limited China’s contributions to global health, and also make it difficult to meet the high expectations of the international community.

It is widely recognized that the UK has been one of the leading countries to promote global health. The UK has helped set up guidelines for global health governance, providing intellectual products relating to global health, rendering DAH, and maintaining national and global health security, all of which China can learn from [22–26], especially its far-sighted and fruitful health diplomacy, such as the establishment of the Department for International Development (DFID) in 1997 and the publication of its first national Global Health Strategy in 2008 [27–29]. Since 2011, the UK has been transforming its relationship with emerging countries, including China, from an aid-based development relationship into a meaningful and mutual partnership for global development [30].

Under this circumstance, the governments of China and the UK signed a memorandum of understanding (MoU) in 2011 to promote international development cooperation, identifying global health as a new field for further strategic cooperation between the two countries. On September 17, 2012, the Ministry of Commerce of P.R.C. and the DFID of the UK formally signed the MoU on the *Global Health Support Programme* (GHSP).

## Programme design and implementation

### Programme design framework

The GHSP was implemented between October 2012 to March 2019 with about 12 million pounds financed by the UK. Its aims were to explore a new type of China-UK partnership, to strengthen their cooperation in global health, enhance China’s capacity to engage in global health governance, and provide effective DAH, in order to jointly improve global health outcomes.

**Fig. 1:**
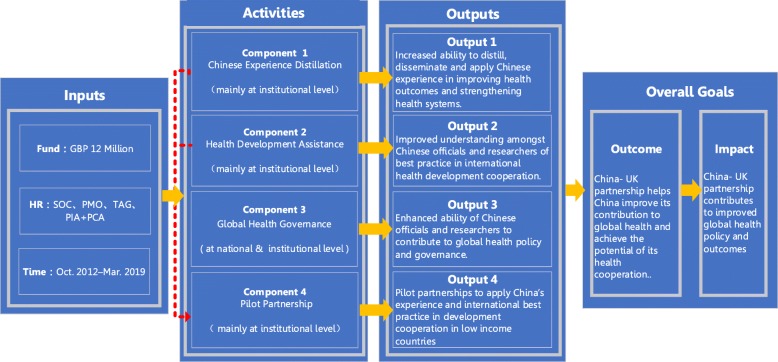
The logic diagram of the GHSP

The GHSP was programmed to support capacity building activities in four components, so as to achieve the following four outputs: (1) Increased ability to distil, disseminate and apply the Chinese experience in improving health outcomes and strengthening health systems; (2) Improved understanding amongst the Chinese officials and researchers of best practice in international health development cooperation (including bilateral and multilateral); (3) Enhanced ability of Chinese officials and researchers to contribute to global health policy and governance; (4) Pilot partnerships to apply China’s experience and international best practice in development cooperation in low income countries. The activities of the programme were carried out at national and institutional levels. In addition, the outputs of the activities in “Component 1” and “Component 2” provided significant support for the design and implementation of the activities in “Component 4”. The logic diagram of the GHSP is shown in Fig. 1.

### Design of programme activities

At the national level, the programme emphasized the concept of “Strategy First” and focused on building platforms and setting up mechanisms. At the institutional level, the GHSP was expected to engage in “Learning by Doing” to build capacity, emphasizing the “Multi-disciplinary, Cross-sectoral and Trans-regional” concept of global health, with the focus on building bridges between research and decision-making. The programme activities were designed to improve five dimensions of capacity: research and analysis, dissemination and training, policy consultation, overseas practice, and pilot partnerships. The main types of activities are shown in Table 1.

**Table 1:**
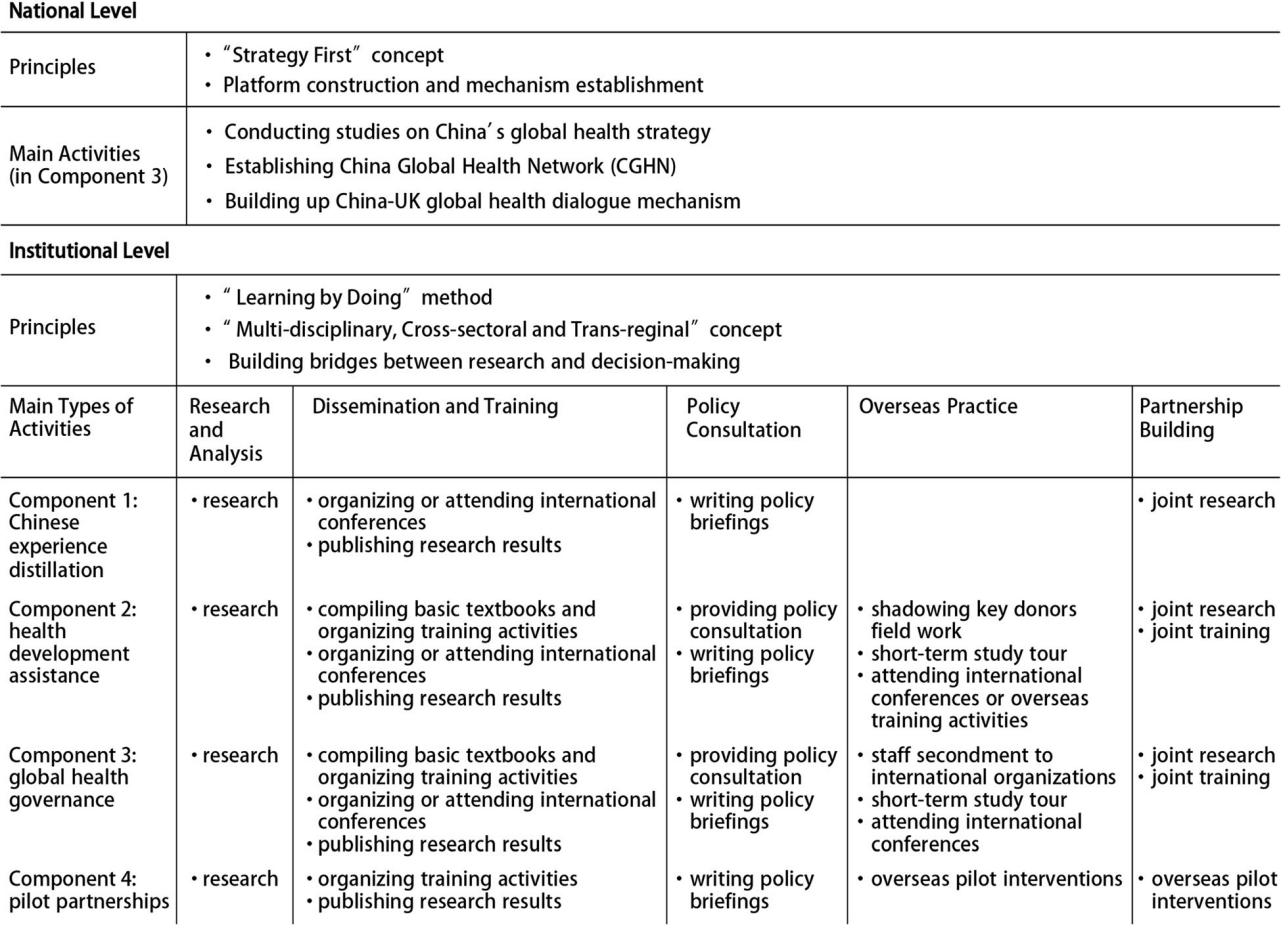
The design of GHSP activities

### Organizational Structure

**Fig. 2:**
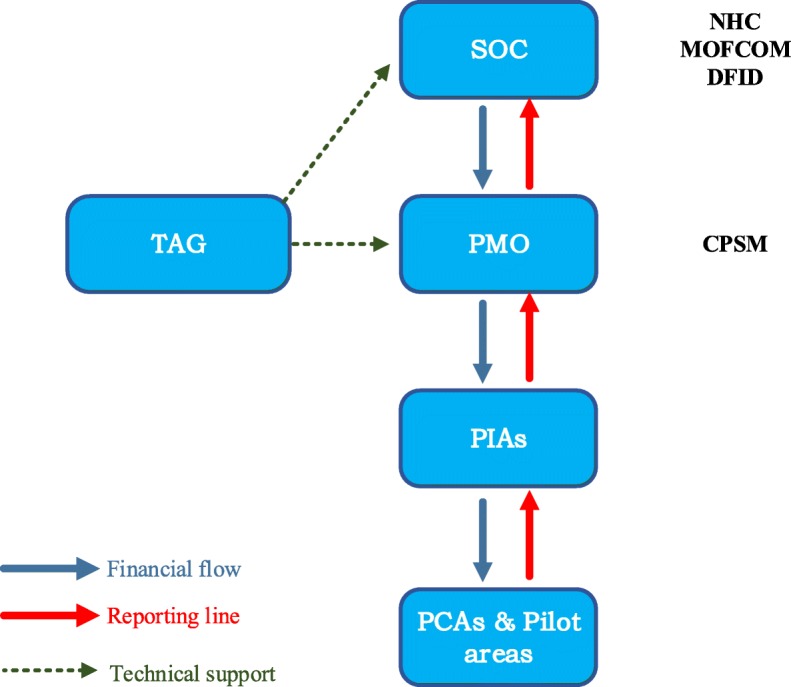
Organizational structure of the GHSP

Figure 2 shows the GHSP’s organizational structure. The strategic oversight committee (SOC) of the GHSP was composed of delegates from the China’s National Health Commission (NHC), China’s Ministry of Commerce (MOFCOM) and the UK’s DFID. The SOC was responsible for setting programme priorities, approving annual work plans and budget, reviewing progress reports and assessing ongoing performance, as well as overseeing the monitoring and evaluation. The project management office (PMO) was located in the Center for Project Supervision and Management (CPSM) of NHC and was responsible for its daily operation and management. GHSP’s technical advisory group (TAG) was composed of independent individual consultants and the WHO China Office, which is responsible for providing the SOC and the PMO with technical advice and support. The technical activities were organized and implemented by different project implementing agencies (PIAs) and their project cooperative agencies (PCAs) or pilot areas.

### Programme implementation

#### Programme management rules

All programme management rules were detailed in the *Programme Management Manual (PMM)*, which is one of most important documents of the GHSP. It was drafted by the PMO at the beginning of the GHSP, approved by the SOC, and adhered to by all programme stakeholders, irrespective of the institution or individual, throughout the whole implementation cycle. The PMM was developed in accordance with the relevant China and UK laws and regulations, the donor’s (the DFID) special requirements, all programme legal documents, as well as the international project management norms. It has clearly defined the organizational structure and responsibilities of each component, detailed rules and regulations for daily management, financial management, procurement management, monitoring and evaluation.

#### Programme decision-making mechanism

The SOC meeting involved decision making and was held twice a year. The main topics included: reviewing and approving the annual work plan, reviewing the progress, discussing important issues and possible solutions. During its intersessional period, if there were any important issues that needed the collective decision-making of SOC, the PMO could propose an ad hoc SOC meeting or communicate with SOC members by email, depending on the complexity of the issue.

#### Selection and determination of the institutions and individuals involved in GHSP

The SOC member units were jointly nominated by China and the UK during the project preparation period. Other related institutions and individuals participating in the GHSP were selected as follows:

1. The PMO was selected by the SOC through bidding, after which the DFID China office and the PMO signed the *GHSP Project Management Agreement*;
2. The TAG members were selected by the SOC’s collective discussions based on the list of international and domestic candidates, which were nominated by SOC members separately according to the requirements of the Terms of Reference (TOR) approved by all SOC members. The TAG members’ contracts were first signed and managed by the DFID China office, and later the job was taken over by the PMO.
3. PIAs. A PIA for each task was selected through bidding or direct selection. Bidding was frequently adopted in the early stage of the programme. The direct selection had been mainly used in the later stages of GHSP for seeking a qualified PIA, which was first nominated by the SOC under any of the following three circumstances: (a) The task was awarded a small amount of money; (b) The task was a natural extension of the previous one; (c) A task that only one institution could be qualified to carry out.
  The TOR of each individual task was jointly drafted by the PMO and the TAG members based on the programme design documents and the approved annual work plan. This was then finalized after the SOC’s approval. The PMO was responsible for both the bidding process and managing the consulting service contracts with each PIA.
4. PCAs. The GHSP was committed to promoting cross-border communication and collaboration, attracting as many institutions as possible. For large-scale tasks, PIAs were thus encouraged to invite domestic or international partners for joint bids. After winning the bid, the joint bidding agencies became the PCAs on this task, and the PIAs would start the work with their PCAs by signing contracts or agreements with them.

#### Programme quality control

The quality control of the GHSP included five main aspects:

1. Logical framework approach. A logical framework with expression of the GHSP’s result chain was formulated by the design team prior to the official launch of the GHSP. It was fundamental to monitoring the progress and evaluating the results of the programme. In accordance with the GHSP’s requirements, the PMO updated data of the logical framework indicators every year to indicate whether or not the programme was going as planned.
2. Annual work plan approval. At the beginning of each year, the PMO and PIAs formulated their annual work plans according to the programme design documents, the logical framework and individual task proposals. Annual work plans were taken as the basis for activities and payments, which had to be reviewed and approved by the SOC in advance.
3. Process review. The reviews mainly consisted of payment reviews conducted by the PMO, semi-annual progress reviews and on-site supervisions on pilot areas by the SOC, annual audits by the China National Audit Office, and annual reviews by the special experts designated by the DFID. The process review paid special attention to the following dimensions: implementation progress, logical framework indicators, the quality and quantity of the intermediate outputs, and the compliance with financial and procurement requirements.
4. Completion acceptance of each task. The PMO carried out the completion acceptance for each task before the last payment, which was used to confirm the expected outputs, the compliance with the financial and procurement requirements, as well as the total final accounts. In order to achieve the “Money for Value” proposed by the DFID, the PMO opted to manage this programme with the method of “linking payments with the quality and quantity of the outputs”, which ensured that each PIA would be able to submit qualified outputs as planned.
5. Independent evaluation. GHSP adopted a third-party evaluation to comprehensively assess its implementation progress and final outputs and achievements. This was composed of a baseline survey, and mid-term and final evaluations. The independent evaluation team was selected by the DFID through international competitive bidding at the initial stage of the GHSP. Evaluation reports written by the independent evaluation team of each stage provided evidence for the SOC to determine the work priorities at the next stage, as well as evidence for the Chinese and British governments to explore future cooperation.

## Main achievements of the GHSP

In its nearly 7 years of implementation, the GHSP achieved its original goal, i.e. it helped build up a sustainable development partnership between China and the UK, which has great potential to facilitate continuous exchanges and cooperation between the two countries, and to contribute to improved health policy and outcomes globally. It is delighted that the outputs were even better than expected (Table 2).

**Table 2:** GHSP logic framework indicators achievements

Output Indicators	Target	Actual
Indicator 1.1 Number of Chinese individuals and institutions supported by GHSP with strengthened capacity to distill Chinese experience in improving health outcomes and strengthening health systems.	34 individuals + 10 institutions	98 individuals + 11 institutions
Indicator 1.2 Number of publications supported by GHSP disseminating Chinese experience in improving health outcomes and strengthening national health systems in a way that is relevant to LMICs.	200	275 (87 research reports + 126 journal papers + 48 policy briefings + 14 published books)
Indicator 1.3 Number of research partnerships between Chinese and LMIC institutions	10	26
Indicator 1.4 Number of research dissemination events with low-to-middle income countries (LMIC) partners, to include researchers and public health officials	10	18
Indicator 1.5 Chinese institutions develop capacity to use evidence on clinical effectiveness to make proposals for policy and clinical guidelines to improve allocative efficiency in the health sector.	2	4
Indicator 1.6 Chinese institutions share experience of improving allocative efficiency with LMICs.	2	4
Indicator 2.1 Number of policy or programmatic papers on development cooperation in health	15	67
Indicator 2.2 Policy- and project- relevant research papers developed reflecting international practice in development cooperation in health (DCIH)	20	35
Indicator 2.3 Core Chinese institutions developed with capacity as think tank and training provider in development cooperation in health	4	6
Indicator 2.4 Development of a cadre of Chinese consultants supported by GHSP with capacity to support Development Cooperation in Health and actively engaged in support to the Chinese government, global health institutions, LMICs governments and/or agencies (Consultants supported by the programme providing support for government)	50	135
Indicator 3.1 Establishment and strengthening of China Global Health Network, providing a forum for discussion, development and mutual learning among PIAs and other concerned institutions	Establishment and functioning of the Network	The CGHN was established in Dec. 2015 and is still operative
Indicator 3.2 Policy-relevant research produced and proposals developed for a China’s global health strategy	10 researches + 1 proposal	11 research studies + 1 proposal
Indicator 3.3 Increased collaboration between China and UK through High Level Global Health Dialogue	Joint work on global health issues	Five dialogues convened regularly on key global health issues of mutual concern; A joint visit to Ethiopia on Africa CDC related issues in 2018
Indicator 4.1 Number of pilot partnerships implemented	2	4
Indicator 4.2 Pilots incorporate China experience and international practice in DCIH.	2	4
Indicator 4.3 Pilot partnerships lead to improved Chinese engagement in global health cooperation	2	4

Data source: *GHSP project completion report* from PMO, Mar 2019

### National Level

#### China-UK Global Health dialogue

The GHSP has supported five rounds of China-UK global health dialogues since 2013. This is the first dialogue on global health between China and a major Western country. The China-UK global health dialogues were held between director-general level officials of the relevant departments of both sides. The establishment of this mechanism enabled two global health powers to complement each other and form synergies, thus more effectively contributing to global health governance, ultimately improving global health outcomes and promoting the achievement of sustainable development goals (SDGs). Depending on the status of the domestic and international health development and the concerns of both sides, the focus of each of the dialogues varied. The topics discussed in each dialogue are presented in Table 3.

**Table 3:** Topics discussed in China-UK Global Health Dialogues

Time	Venue	Topic discussed
First Dialogue Mar. 11, 2013	London, UK	Universal health coverage (UHC), access to medicines in the context of malaria and poliomyelitis control, international health policy and governance, and post- MDGs and health, etc.
Second Dialogue Nov. 12, 2014	Shanghai, China	Access to essential drugs, Ebola response, reproductive, maternal and child health (RMNCH), international health partnerships and governance, post MDGs and health, and China global health strategy, etc.
Third Dialogue Sept. 14, 2015	London, UK	Antimicrobial resistance and drug resistant malaria, post-Ebola collaboration, health and SDGs, and WHO reform, etc.
Fourth Dialogue Jul. 12, 2017	Beijing, China	WHO reform agenda, policy update, health cooperation in Africa, second phase of global health collaboration, etc.
Fifth Dialogue Jan. 22,2019	London, UK	New global health programme design and development, WHO reform, UHC, collaboration on global health security in Africa, etc.

Data source: GHSP *project completion report* from PMO, Mar 2019

#### Studies conducted on China Global Health Strategy

**Fig. 3:**
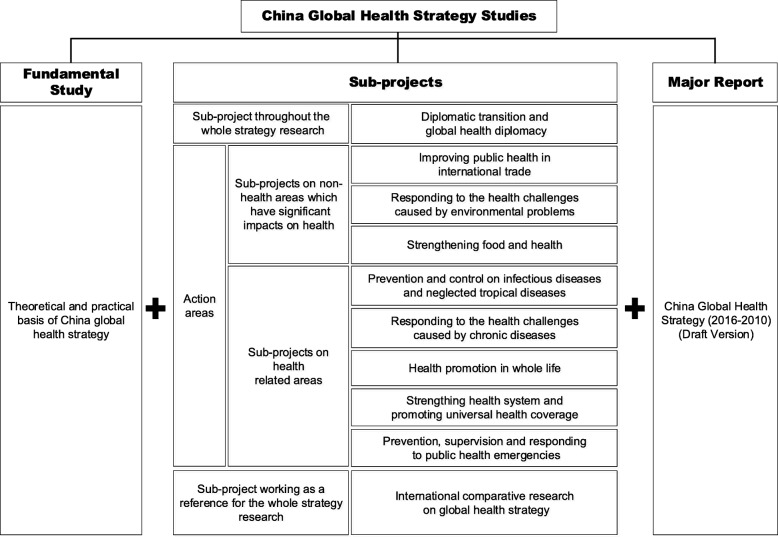
Series of studies on China global health strategy

The GHSP promoted a comprehensive set of studies on global health strategies, producing a total of 12 research reports and a draft of China’s global health strategy, which provides data, facts and constructive policy recommendations for the central government of China to develop its national global health strategy document. The topics of these studies are shown in Fig. 3.

The research recommendations on China’s global health strategy are consistent with the *Healthy China 2030 Plan* and *13th Five-year Health Plan*, released by the central government of China in 2016. *Health China 2030 Plan* includes assertions such as “China’s global health strategy will be implemented”, “By establishing high-level strategic dialogues between countries, China will encourage putting health on the diplomatic agenda of major countries”, “China will actively participate in global health governance, gain international influence and a strong voice in building institutional health”, and so forth [31]. The *13th Five-year Health Plan* includes assertions such as “develop China’s global health strategy”, “improve China’s influence and strengthen China’s leading voice in global health diplomacy”, “continue to strengthen health assistance to foreign countries”, and “promote global health personnel training and team building” [32]. These statements highlight how the relevant research work of GHSP has gained positive affirmation from the China’s NHC and also the top decision-makers of the central government of China.

#### The establishment and operation of the CGHN

**Fig. 4:**
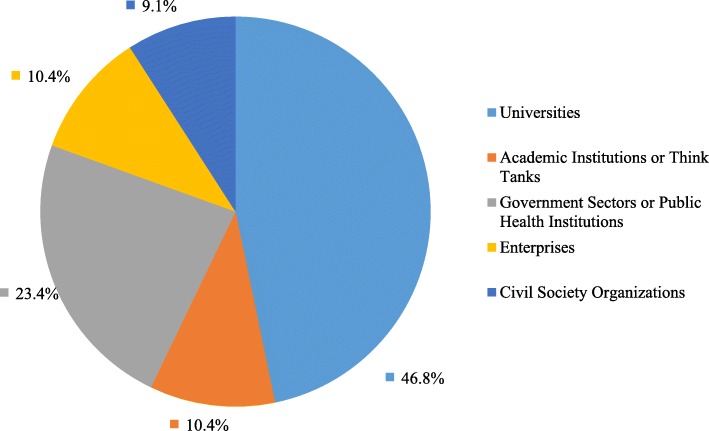
Categories of the CGHN members. Data source: The CGHN secretariat office report, May 2019

With the support of GHSP, the China Global Health Network (CGHN), a non-profit, membership alliance was founded in Beijing on 6 December 2015. The School of Public Health, Peking University was elected as the first round of leading organization of the CGHN, and was responsible for the CGHN secretariat. The CGHN adheres to the principle of openness and inclusiveness, as well as welcoming various actors to join in the network and participate in relative activities. As of March 2019, organizations that joined the CGHN increased from 46 at the beginning of its establishment to 77. They are universities, academic institutes, think tanks, governmental units, public health institutions, as well as enterprises and civil society organizations in more than 20 provinces and municipals (Fig. 4). The CGHN plays a key role in expanding and consolidating the influence of GHSP and promoting China’s global health cause.

Since its establishment, the CGHN has not only functioned a global health communication platform for network members, but it also became an exchange channel between China’s global health community and the external world. This is done through co-convening international conferences, carrying out studies commissioned by health departments on global health cooperation, developing capacity-building training activities, and establishing partnerships with overseas institutions.

### Institutional level

A priority of GHSP is to improve the ability of Chinese institutions to participate in global health. During the seven-year period, 53 Chinese institutions, either as PIAs or PCAs, were involved in various project activities. GHSP has promoted a number of universities and research institutions to become the backbone for global health research and practice in China, such as the six Centers of Excellence of the GHSP, namely, Peking University School of Public Health (PKUSPH), Fudan University School of Public Health (FUSPH), Fudan University Global Health Institute (FUGHI), Center for Global Public Health of China CDC (China CDC), National Institute for Parasitic Diseases of China CDC (NIPD), and China National Health Development Research Center (CNHDRC).

#### Research and analysis

**Fig. 5:**
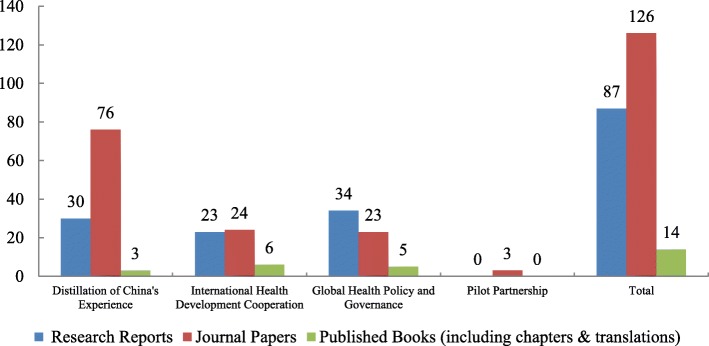
Research outputs of GHSP by activity components. Data source: GHSP *project completion report* from PMO, Mar 2019

PIAs carried out a series of policy studies on the distillation of the Chinese experience, international health development cooperation, global health policy and governance, and pilot partnerships in other developing countries, thereby improving their expertise in relation to global health research and analysis. The outputs generated by these PIAs have been published in the form of research reports, papers and monographs, etc. through domestic and international publishing houses and academic journals. Figure 5 shows the number of different types of GHSP academic outputs generated under the four components. As of March, 2019, the programme had produced 87 research reports, 126 journal papers (57 in English, 69 in Chinese) and 14 books (including chapters and translations). See Additional file 2 for more details.

#### Dissemination and training

The GHSP enhanced the skills of Chinese institutions in terms of dissemination and training, for example in supporting PIAs to develop training materials and basic textbooks on DAH and global health governance; organizing short-term training courses, participating in international exchange activities, and facilitating domestic English academic journals in global health, etc. In total, the programme facilitated 109 international exchange activities involving 349 professionals. Those activities included attending international conferences, sending experts to a few developing countries to provide consultation services; and sending young researchers to international research institutions or universities for short-term study or training (see Additional file 3 -1 for details). The GHSP assisted PIAs in organizing 27 global health training and practice activities, totaling 1020 person-times (see Additional file 3-2 for details). Participants included government officials, researchers, public health practitioners and managers of international cooperation projects.

The GHSP also played a key role in nurturing talent in relation to global health in the Chinese universities. For example, Peking University set up two postgraduate courses (*Introduction to Global Health* and *Global Health Governance*) on the basis of textbooks developed under the GHSP; Fudan University developed the online MOOC course “Introduction to Global Health”; and through a cooperation with the GHSP, *Global Health Research and Policy*, a professional English-language academic journal launched and operated by Global Health Institute of Wuhan University, enhanced its own international influence by helping to expand the international dissemination of several GHSP outputs.

#### Policy consultation

**Fig. 6:**
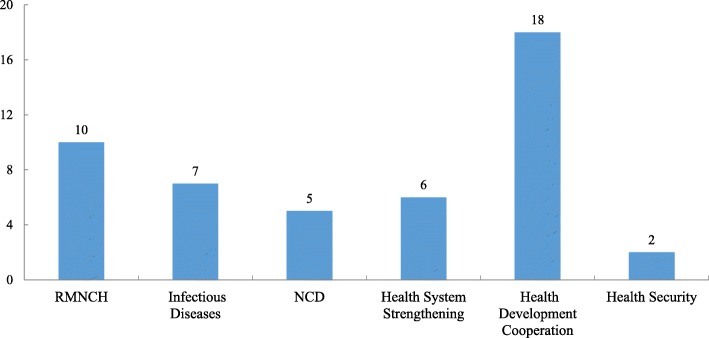
Topic distribution of GHSP policy briefings. Data source: GHSP *project completion report* from PMO, Mar 2019

Under GHSP’s support, PIAs provided the governments of China and a few other developing countries with a total of 48 policy briefings in both English and Chinese, which were written on the basis of the policy research results and focused on significant global health issues. Figure 6 shows the topics of the policy briefings, and Additional file 4 gives their titles. A number of policy briefings also drew the attention of the Institute of Information Studies under the Chinese Academy of Social Sciences, and were modified and submitted to the highest decision-making level of the central government of China in the form of internal reference materials.

PIAs also provided professional expertise and consultation to the central government of China and international organizations by: (1) participating in consultation activities and drafting a number of global health documents, such as *China Global Health Strategy, Healthy Asia-Pacific 2020 Initiative, China Belt and Road Initiative Health Exchange and Cooperation Implementation Plan (2015–2017&2018–2020), China Assistance in Building 100 Medical Institutions Plan, Cultivating Health Talent Programs in Developing Countries* and *Healthy China 2030 Plan;* (2) giving advice to the delegation of China NHC during the WHO Executive Committee Meeting and the World Health Assembly; (3) serving on the technical committees of international organizations such as WHO, and providing advice on maternal and child health, the prevention and control of malaria and tropical disease, etc.

#### Overseas practice

The GHSP supported one malaria control pilot project in Tanzania, two maternal and child health pilot projects in Myanmar and Ethiopia (see Additional file 5), and other related overseas capacity building activities. For example, assigning a few cadres to some international organizations on secondments, assisting Sierra Leone to enhance its public health surveillance capacity, and conducting consultations and co-sponsoring seminars with international public health partners who are working in Africa. The above activities have improved the ability of Chinese institutions to act overseas in three ways:

1. By applying the Chinese experience to other developing countries, PIAs tried to tailor the Chinese experience to the local environment in the design and implementation process, which helped improve the projects’ applicability and contributed to achieving the expected objectives. For example, based on China’s “1–3-7″ model on malaria elimination, a community-based 1, 7-mRCT model was established in the malaria control pilot. The new model rapidly reduced the malaria burden in the pilot areas of moderate and high transmission in Tanzania with a verified decline in the prevalence of malaria of over 70%. The maternal and child health pilot adopted the typical Chinese “three-ring strategy”, which required finding a linker to connect the demand, supply and payment together. In China, traditional family midwives were trained to take the responsibility as a linker. While in Ethiopia and Myanmar, depending on their situation, pilots selected health extension workers (HEW) and assistant midwives (AMW) to play the role as linkers respectively, whose efforts significantly increased the provision of RMNCH services in the pilot areas. In Ethiopia, the institutional delivery rate improved from 28 to 55% within the project period, and the same indicator also saw an increase in Myanmar from 30 to 53%.
2. By managing health development cooperation projects. There are large differences between the pilot countries and China in terms of political and economic systems, social structures, languages and culture etc. The overseas pilot projects also commonly last for a long time period, involving a large amount of funds and many stakeholders. All these factors entail potential risks and uncertainties in terms of project management. Consequently, the Chinese PIAs continuously learned from practice, accumulated experience, and gradually improved their professional expertise through cooperation with local partners. Meanwhile, confronted with the China’s current mismatch between the policies and the need for “going out”, Chinese staff exercised their ability to actively seek solutions to achieve the pilot’s objectives.
3. By cooperating with the international health community. Through the GHSP, Chinese institutions were able to closely observe and learn how international communities work. For example, the programme enabled officials from the NHC of China to participate in an on-site joint study tour in Africa with expert groups from the UK and WHO. It also supported seven Chinese cadres to work on secondment at the WHO and other international organizations. Chinese institutions and individuals have gradually recognized the importance of maintaining close ties with all partners from the international community and have improved their communication skills with the external world. For example, the Chinese public health team in Sierra Leone established technical communication mechanisms with local government, partners from the UK, the US and local branches of international organizations to share work results over time.

#### Partnership building

**Fig. 7:**
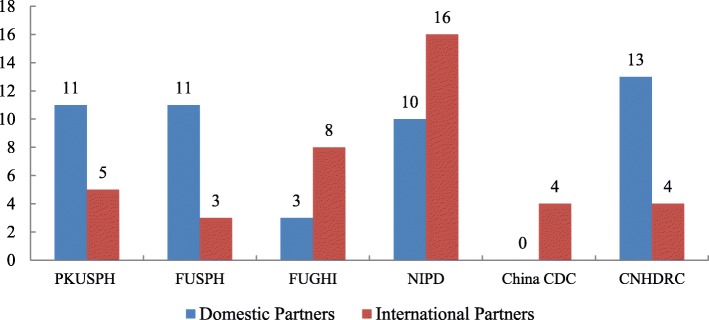
Partnerships established with six Centers of Excellence under the GHSP. Data source: The GHSP *project completion report* from PMO, Mar 2019

During the implementation of GHSP, six Centers of Excellence established stable partnerships with a number of domestic and international institutions (Fig. 7). Domestic partners included health-related governmental departments, universities, research institutions, hospitals and pharmaceutical companies. International partners included international organizations, universities, professional research institutes and other civil society organizations in over 20 countries. Through the GHSP, PIAs have enhanced their expertise by learning from each other thereby achieving mutual benefits. Besides academic achievements, PIAs have also improved their managerial skills in support of the common goal of cross-border, trans-cultural and multi-disciplinary teams. In addition, pilot projects have boosted the mutual understanding among the governments of China, the UK and the pilot countries as well as other stakeholders.

## Reflections and policy implications

### The necessity of good programme design to ensure the achievements of expected goals

The GHSP had consecutively been scored A or A+ in each of the annual reviews by the DFID. The 2018 annual review report concluded that: “Experience to date suggests that the GHSP represents even greater value for money than was expected in the business case”. In fact, all the successful achievements had benefited from the good design, which is shown in three features as below.

1. A well-structured design team. The whole design process had lasted for more than 1 year. Composed of experienced experts from the UK, China and Africa, the design team had a comprehensive understanding of the policies and practices of international development assistance and British development cooperation, the challenges of China’s participation in global health, and the requirements of health development in Africa. The design team provided their expertise for the design of GHSP and ensured its rigor and rationality as much as possible. In order to maintain the capacities acquired through the GHSP in line with the actual needs of developing countries, the design team consulted relevant domestic management personnel and experts in China and took a field trip to Uganda. They visited government departments and senior leaders, professional institutions, and major local international partners. They also visited China’s health aided sites, such as local hospitals assisted by China, and the China medical team working there.
2. Accurate problem identification. The design team identified three main global health capacity insufficiencies concerned with China prior to the design.
  1. Although the Chinese academic community has conducted a number of studies on China’s health development experience widely recognized by the international community, these studies lack the perspective of external applicability and are not fully reflected in the global pool of knowledge.
  2. Although China has a long history in providing DAH to developing countries, the main types of DAH are limited to sending medical teams and building health infrastructures [33, 34]. China lacks understanding of the practices provided by other DAH actors, particularly the best practices of contemporary international DAH. Therefore, China urgently needs to learn from the mature experience of some Western countries, improve its ability to implement public health interventions for serious diseases and other major health issues in developing countries, and enhance its ability to coordinate actions with other development partners.
  3. China has a strong will to actively participate in global health governance and contribute to global health solutions with China’s wisdom, especially in studying, negotiating and formulating relevant international standards, norms, guidelines, etc., however, China still lacks the capacity to participate in global health governance and policy development.
3. Logical correlations established among different activities.

In order to solve the problems identified by the design team, the GHSP’s activities in Components 1–3 were proposed to enhance capacity in Chinese experience distillation, DAH, and global health governance respectively. Component 4 was designed to set up partnership pilots, i.e. applying China’s experience distilled under Component 1 and international best practices of DAH learned under Component 2 to one or two selected Asian or African countries. Therefore, Component 4 could be considered as an experiment of the results of Component 1 and 2. In addition, the enhanced capacity at national level under Component 3 was also expected to provide policy support for the pilot projects in Component 4. It turned out that the rational interaction among different components could be helpful to achieve the final goals.

### Building up sustainable development partnerships through pragmatic cooperation

“Strengthen the means of implementation and reinvigorate the global partnership for sustainable development” is one of the important goals of *The United Nations 2030 Agenda for Sustainable Development*. The Agenda states that, “we will not be able to achieve our ambitious goals and targets without a revitalized and enhanced Global Partnership”. The specific statement of the capacity building goal in SDGs is to “Enhance international support for implementing effective and targeted capacity-building in developing countries to support national plans to implement all the sustainable development goals, including through North-South, South-South and triangular cooperation” [35]. In this regard, the GHSP provided a good example, particularly in the following two aspects.

Firstly, the GHSP explored a specific path to transform “North-South Aid” to “North-South Cooperation”. While China and the UK represent the South and North respectively in many typical ways, the GHSP has transformed the traditional goal of DAH from “the development of the recipient countries” to “improving the contribution of the recipient countries to global health”, to which the approach was also transformed from the “providing financial support” to “building up long-term cooperation partnerships”. Therefore, the bond between the two countries was successfully transformed from “Aid” into “Cooperation”.

Specifically, the GHSP partnerships were established at two different levels:

1. Global health policy dialogues at the national level. The GHSP established regular high-level dialogue between China and the UK. This facilitated the exchange of views on key current global health issues and increased mutual understanding between the two governments. It also provided a platform for seeking consensus on major health issues in global health and for exploring collaboration in global health governance.
2. Technical cooperation at institutional level. The GHSP facilitated institutions and individuals from both China and the UK to take joint actions, such as overseas pilots in Asian and African developing countries, flagship training workshops on DAH, global health policy research, and managing projects under trans-cultural environment. In addition to close contacts between the two governments, the Chinese PIAs established ties with more than ten British institutions (academic institutes, think tanks and civil society organizations). Those relations have evolved into substantial partnerships, and the outputs generated through the partnerships have become public goods, contributing to the international community and other developing countries.

In fact, during the implementation of the GHSP, China-UK cooperation had not only entailed discussing principles, exchanging opinions and reaching basic consensus, but more focused on pragmatic and down-to-earth actions, such as holding regular dialogues at a national level and conducting joint activities at an institutional level. These nurtured the authentic transformation from initially being total strangers to becoming closely-knit partners with a mutual understanding. The partnership created in the process would be helpful to contribute to the sustainable development. The GHSP’s “North-South Cooperation” could be a model for cooperation between the UK and other emerging countries, and provide references to further cooperation between China and other western countries in both health and other development fields.

Secondly, the GHSP explored a model for triangular cooperation in global health. Triangular cooperation in development assistance, traditionally speaking, refers to cooperation among three sides: a developed country (or international organization) that provides funds and has rich experience in traditional development assistance, a developing country with certain knowledge and capabilities (such as China and India), and another developing country or a group of developing countries who receive development assistance [36].

An increasing number of stakeholders believe that triangular cooperation is not only a useful channel to connect “North-South Aid” and “South-South Cooperation”, but also useful to enhance the effectiveness and efficiency of development cooperation. In the past, China has carried out triangular cooperation in non-health fields with international organizations. However, there have been very few cases of triangular cooperation between China and developed countries in health before the launch of the GHSP. The GHSP has supported three overseas pilots with funding, in which China, the UK and the PCAs of pilot countries jointly determined the pilot themes, while the PIAs of China and the PCAs of the pilot countries were responsible for the project design and implementation, and the UK provided technical support and management guidance as needed.

The ultimate success of the overseas pilots can be attributed to the organic triangular cooperation, i.e. funding and consulting from the UK, technical and practical skills in RMNCH and disease control, and development experience from China, along with the willingness and efforts of the PCAs of the pilot countries.

However, there are several limitations. First, the triangular cooperation had involved many stakeholders with different concerns, which consequently increased the time and communication costs. Second, in the pilots, although the PIAs had respected the will of the host countries, the local or national governments of those countries were not fully involved in the cooperation because the initiators of the triangular cooperation were China and the UK, while the pilot projects were designed by the PIAs of China and the PCAs of the pilot countries rather than the local governments. A number of actions had to be taken to fix this deficiency in the later stage. Therefore, it is suggested that the communication and engagement of local government should be fully considered in the initial stage in future triangular cooperation.

### Extending global health engagement through multi-sector reforms

When the GHSP began, China’s knowledge, research and training capacity in relation to global health were still in its infancy stage. Few policy makers and professionals in the health sectors paid continuous attention to contemporary global health issues. Thanks to the implementation of the GHSP, global health concepts and theories have been widely disseminated in China’s health sector. GHSP-funded policy research activities, especially the strategy research, have enabled the Chinese government to engage in global health governance with a clearer vision and better mission, and to improve the approaches of DAH.

The GHSP has implemented health intervention pilots in three Asian and African countries, exploring new models for China’s health development cooperation, which is a breakthrough and provides valuable lessons for China’s DAH. For example, pilots have explored how to set up public health projects in developing countries, how to improve the effectiveness of DAH through triangular cooperation, and how to coordinate with various local civil society organizations (including international NGOs working locally). During the implementation of the programme, the GHSP managers and the PIAs encountered difficulties as well. In the process of solving these problems, both the GHSP managers and the PIAs gained a deeper understanding of some bottlenecks affecting China’s global health participation and the directions for future efforts.

Firstly, relevant system and mechanisms are urgently needed to be developed to match the emerging needs for new type of DAHs. In recent years, the Chinese government has made many commitments on the global health, such as cooperating with countries and international organizations who have welcomed the Belt and Road Initiative, supporting the African CDC, and launching programmes to deal with new and re-emerging infectious diseases, preventing and controlling schistosomiasis, AIDs, and malaria in Africa, etc. [37, 38]. In order to fulfill these commitments, new and innovative means for providing DAH are required.

To ensure that the Chinese health experience and its technical advantages can be fully exerted in other developing countries, the following three issues need to be addressed:

1. An innovative system for “going out”. There is a lack of supporting mechanisms and policies for domestic institutions and personnel “going out” in foreign exchange control, rules for public institutions regarding overseas trips, exit-entry administration, the legal status and remuneration of personnel, staff health and security, and insurance policies, etc.. In fact, some current policies and regulations are not fully able to meet the actual needs of staff working overseas for a long-term, which for example, delayed working progress, increased management costs, and even posed much higher risks to health workers. These problems cannot be solved by the PIAs themselves, and require national coordination. Therefore, many of the policies and regulations need to be updated.
2. More comprehensive selection of “going out” institutions. The PIAs of the GHSP overseas pilots mainly came from universities and public health institutions. They were characterized by strong technical advantages but with relatively insufficient management capacities. With the rising demand for “going out”, in addition to universities and public health institutions, China also needs to learn from the general practices of the international community and provide more opportunities to domestic civil society organizations that have a comparatively high level of internationalization and sustainable “going out” capabilities for overseas engagement.
3. More awareness and expertise on cross-cultural environments. Although the GHSP overseas pilots achieved the expected results, the process had many twists and turns. In the early stage, the pilots progressed slowly, partly because the Chinese PIAs lacked professional management skills and a good understanding of international management norms, and therefore were unable to manage risks. In fact, project management skills are exactly the key to ensure effective utilization of resources in DAH. A long-term plan should therefore be developed for the gradual improvement of project management. It also suggested that China should intentionally learn project management practices from traditional donor countries or international organizations through triangular cooperation.

Secondly, global health governance requires multi-sectoral involvement. The experience of the international community, especially the UK and other leading countries, shows that global health requires full-scale coordination and the advocacy of stakeholders from the whole society. It is definitely not the sole responsibility of health authorities. Although the GHSP has played an important role in raising the awareness of China’s health sector in the concept of global health, the relative advocacy for other governmental departments (e.g. the Ministry of Foreign Affairs, the Ministry of Finance, the General Administration of Customs) has been very limited. The *Healthy China 2030 Plan* issued in 2016 clearly stated “Health in All Policies” [31]. The *Healthy China Action (2019–2030)* released in July 2019 also clarified specific responsibilities of various departments [39]. These government documents have laid a solid foundation for China’s multi-sectoral participation in global health governance. More actions need to be taken to encourage information sharing, negotiation and coordination among all governmental departments regarding global health issues, and to continuously develop and publish a whole of government global health strategy. In 2018, the Chinese government set up the China International Development Cooperation Agency (CIDCA), which is helpful not only to integrate health issues into the DAH domain, but also to formulate China’s global health action strategies, establish relevant mechanisms, and introduce future national policies that support these strategies.

### Ample room for improvement in global health research and policy consultations

The development of relevant policies on global health requires a large amount of evidence-based knowledge and analysis. Therefore, the GHSP focused on improving researchers’ ability to provide a high-quality policy consultation. On the one hand, the programme encouraged them to build close ties with decision makers. One typical example was the research on China’s global health strategy. Government authorities were invited to participate in the design of the studies and to review periodical results. As a result, the research team was directly nominated to help the government draft the policy document “China Global Health Strategy”. The close ties between the researchers and decision makers not only facilitated the researchers’ understanding of the actual needs of the government, but also increased the governmental departments’ recognition of their research outputs, thereby catalyzing the transformation of research outputs into policies. In fact, shortly after the completion of the strategic research activities, the proposed “China Global Health Strategy” was also officially introduced as an internal policy document.

On the other hand, the GHSP provided professional training and guidance for writing policy briefings to all research teams. Therefore, in addition to the publications in domestic and international publishing houses and academic journals, the main research results were also presented in the form of the Chinese and English policy briefings. These policy briefings provided high quality information to decision makers, which enabled them to quickly understand the key issues.

Based on the GHSP’s limitations and findings in this regard, three suggestions for future global health research and consultation are recommended:

1. Both researchers and decision-makers are equally important in evidence-based decision-making. However, the GHSP mainly focused on “improving the researchers’ abilities to provide information and policy consultation”, and ignored the need to improve the decision makers’ abilities to select and use information. In fact, the latter is obviously more important in the process of translating research results into government policies. This aspect should be given more attention to any future similar programme.
2. Researchers need to realize that their close ties with decision makers bring both benefits and challenges. For example, researchers are expected to take into account the opinions of governmental officials and at the same time maintain the independence of the research team. Furthermore, they need to balance the “idealism” of scientific research and the “realism” of the opinions of decision-makers.
3. Global health-related research needs to be further extended. Although the GHSP funded a large number of studies on different topics, there are two research areas that still need to be further explored.
  1. The external adaptability of the Chinese health experience. The GHSP has made groundbreaking attempts to support studies on international adaptation of China’s experiences in strengthening the health system, RMNCH, and disease control, etc. The final research outputs, however, did not fully meet the expectations due to difficulties in the methodology and implementation. In fact, accurate judgements regarding to what extent China’s experience can be adapted to the international context will become a prerequsite for China’s future DAH. The demand for this kind of research regarding health collaboration between China and other countries are likely to increase, especially giving that so many countries have welcomed cooperation with China through the Belt and Road Initiative.
  2. China’s experiences in using funds from international DAH. The GHSP paid much attention to the research on “China’s previous efforts to provide health assistance to other developing countries”, while there has been little research on “how China has effectively used the funds provided by traditional donor countries and international organizations”. In fact, since its acceptance of World Bank loans to support domestic health projects in 1980s, China has benefited a lot from DAH. It is worth summarizing how China as a developing country has effectively used international DAH, by systematically analyzing the specific practices of both China and international partners in development assistance. For example, what strategies has China taken to ensure the effective use of foreign assistance? What concessions have been made by the international development assistance partners to fit in with China’s context? It is believed that recipient countries will gain constructive knowledge from China’s best practices in relation to development assistance. Further research on the above topics may help provide useful implications for China’s upcoming collaboration with other developing countries, and thus contributing to global health.

## Supplementary information

**Additional file 1.** Chinese version of the full report

**Additional file 2.** The Main Academic Outputs and Deliverables Produced by the GHSP.

**Additional file 3.** International Exchange Activities and Training Activities Supported by GHSP.

**Additional file 4.** The Full List of Policy Briefings Produced by the GHSP.

**Additional file 5.** Overseas Pilot Projects supported by the GHSP.

## Data Availability

Please contact author for data requests.

## Abbreviations

AMR: Anti-microbial resistance
AMW: Assistant midwife
CGHN: China Global Health Network
China CDC: Center for Global Public Health of China CDC
CIDCA: China International Development Cooperation Agency
CNHDRC: China National Health Development Research Center
CPSM: Center for Project Supervision and Management of National Health Commission
DAH: Development assistance for health
DCIH: Development cooperation in health
DFID: Department of International Development
FUGHI: Fudan University Global Health Institute
FUSPH: Fudan University School of Public Health
GHSP: Global Health Support Programme
HEW: Health extension worker
LICs: Low-income Countries
LMICs: Low-to-middle income countries
MDGs: Millennium Development Goals
MOFCOM: Ministry of Commerce
MoU: Memorandum of understanding
MSI: Marie Stopes International
NCDs: Non-communicable diseases
NGOs: Non-governmental organizations
NHC: National Health Commission
NIPD: National Institute for Parasitic Diseases of China CDC
PCAs: Project cooperative agencies
PIAs: Project implementing agencies
PKUSPH: Peking University School of Public Health
PMM: Programme Management Manual
PMO: Project management office
RMNCH: Reproductive, maternal and child health
SDGs: Sustainable development goals
SOC: Strategic oversight committee
TAG: Technical advisory group
TOR: Terms of Reference
UHC: Universal health coverage
WHO: World Health Organization
