# Supplementary material for: China-UK partnership for global health: practices and implications of the Global Health Support Programme 2012–2019
Source: Glob Health Res Policy. 2020 Mar 20;5:13. doi: 10.1186/s41256-020-00134-7 (PMC7083009; doi:10.1186/s41256-020-00134-7)
Supplement: Supplementary file 1 — Additional file 1. Chinese version of the full report [file 41256_2020_134_MOESM1_ESM.pdf]

Additional file 1

中英合作促进全球健康

----中英 GHSP 项目（2012-2019）的实践和启示[1]

王晓华 [1]，刘培龙 [2]，徐彤武 [3]，陈岩 [4]，俞骞 [1]，陈逊 [1]，陈静宜 [1]，张朝阳 [1]

(1.国家卫生健康委项目资金监管服务中心，北京，100044；2.北京大学公共卫生学院，北京，100191；3.中国社会科学院研究生院，北京，102488；4.武汉大学健康学院/全球健康研究中心，武汉，430071)

【摘要】

背景：在过去的几十年中，全球卫生面临着一系列重大挑战，要求中国更为深入地参与到全球卫生治理工作中来。在此背景下中英全球卫生支持项目（GHSP）于 2012 年启动。该项目由英国政府资助，总金额约 1200 万英镑。**目标：**本项目旨在建立中英卫生新型合作伙伴关系，加强双方在全球卫生领域的合作，提升中国参与全球卫生治理、有效提供卫生发展援助的能力，共同促进全球健康状况的改善。**主要成果：**在国家层面，项目支持了 5 届中英高级别全球卫生对话；完成了中国全球卫生战略系列研究，为出台国家相关的政策提供了有力证据；建立了中国全球卫生网络。在机构层面，项目资助了一系列研究、培训、国际交流及试点活动，产出一大批较高质量的全球卫生中英文研究报告和政策简报，培养了一批有志、且有能力参与全球卫生事务的中国机构和人员队伍，同时促进了项目实施机构和合作机构建立伙伴关系，增强了中方机构的境外全球卫生发展援助实践能力，改进了三个亚非国家试点地区的卫生服务提供和健康结局。**政策启示：**中英两国通过在本项目中构建新型双边合作伙伴关系，开展三方合作的实践，切实推进 2030 年可持续发展议程。这种模式为南北合作树立样板，可以为其他国家发展双边关系提供有益参考。

【关键词】 全球卫生政策；全球卫生治理；伙伴关系；中国；英国；卫生项目管理

---

[1] 通讯作者:王晓华，E-mail: wangxh@nhc.gov.cn

1.背景

21 世纪人类深切感受到的一个事实是：在全球治理中，卫生健康相关议题的重要性和紧迫性都空前提升[1]。全球化浪潮的席卷，生态环境的恶化和气候变化的作用[2, 3]，使全球卫生发展和全球卫生安全面临着一系列重大困难和挑战。联合国千年发展目标（MDGs）中部分关键健康指标并没有实现预期目标[4]；全球卫生发展不公平现象日益突出，撒哈拉以南非洲国家的卫生状况尤其令人担忧[5]；非传染性疾病逐渐成为威胁人类生命的主要杀手[6]；具有跨国影响力的重大公共卫生事件，尤其是重大流行性疾病频频发生[7-11]；其他公共卫生安全威胁还包括抗微生物药物耐药性（AMR）的扩散[12]、核生化恐怖主义[13]、极端气候引发的公共卫生危机等[14]。此外，全球卫生发展援助处于转型之中，第二次世界大战结束后形成的国际援助体系亟待改革，援助亟需在满足受援国实际需求，提高有效性、效率、透明度及可持续性方面进行改进[15]。这些困难和挑战绝不是某一个国家可以应对的，需要国际社会所有成员和行动方共同关心、参与、合作应对。中国正是其中不可或缺的重要一员。

首先，中国是全球卫生发展合作的受益者。1949 年中华人民共和国成立以来，以其有限的财力、物力和医疗卫生能力在改善人民健康方面做出了诸多努力[16, 17]。1978 年改革开放之后，中国先后接受了来自世界银行、世界卫生组织（WHO）、抗击艾滋病、结核病和疟疾全球基金、比尔及梅琳达·盖茨基金会等国际机构以及发达国家的大量资金和技术援助。这些支持与中国自身努力有效结合，大大加速了中国卫生健康事业的发展。2011 年中英全球卫生支持项目（Global Health Support Programme, GHSP，以下多采用英文缩写）设计之时，中国已经实现了 MDG 目标四和目标五, 正在努力完成目标六。其次，中国是全球卫生发展合作的贡献者。中国人口众多，约占世界人口的 1/5。中国人民健康状况的改善本身就是对全球健康的重大贡献；此外，中国始终支持全球发展议程，支持世界卫生组织等国际组织的工作，过去几十年间积极开展对外援助和南南合作，力所能及地为其它发展中国家提供卫生方面的帮助[18-21]。最后，中国具备做出更大贡献的潜力。过去 70 年间中国改进自身健康状况过程中积累的大量经验，使得中国具备为全球发展提供更多有益公共产品的可能；近些年，综合国力的快速提升使中国有可能将目光更多地转向全球，中国政府也不断在重大外交场合中对全球

卫生做出了重要承诺；因此，无论是国际社会，还是中国自己的卫生工作者都期待着中国可以为全球卫生做出更多贡献。

然而，与发达国家相比，中国在"有意愿参与全球卫生"和"能够很好参与"之间还是有相当多的不足需要弥补，比如缺少从外部适用性的角度对中国卫生健康经验与教训的梳理；缺乏对国际卫生发展援助最佳实践的了解；缺乏参与全球卫生治理和政策制定的能力；缺乏跨境开展公共卫生干预和联合行动的能力等等。这种状况不利于中国更好地参与全球卫生，并作出更大的贡献，也难以满足国际社会的殷切期待。

在全球卫生领域，英国是公认的领先国家。英国在贡献全球卫生治理规则、提供全球卫生知识产品、开展卫生发展援助、维护本国及世界的卫生安全等方面均具有较为丰富的积累和明显优势，值得中国学习和借鉴[22-26]。特别是其卫生外交深谋远虑，成就斐然，比如在 1997 年建立内阁级的国际发展部（DFID）和 2008 年发布国家《全球卫生战略》[27-29]。英国自 2011 年以来正在力图把同中国等新兴大国的"发展援助关系"转化为对全球发展更有意义的"互惠伙伴关系"[30]。

正是基于上述背景，中英两国政府在 2011 年签署了促进国际发展合作的谅解备忘录，确定把全球卫生作为双方进一步开展战略合作的新领域。2012 年 9 月 17 日，中国商务部与英国国际发展部（DFID）正式签署《中英全球卫生支持项目谅解备忘录》，开展 GHSP 项目。

2. 项目设计与实施

2.1 项目设计

2.1.1 总体设计思路

GHSP 项目是中英两国政府合作开展的一个探索性卫生发展合作项目，2012 年 10 月至 2019 年 3 月实施，总金额 1200 万英镑，全部由英方资助。项目总目标为：建立中英卫生新型合作伙伴关系，加强双方在全球卫生领域的合作，提升中国参与全球卫生治理、有效提供卫生发展援助的能力，共同促进全球健康状况的改善。

项目设计了四个领域的能力建设活动：中国经验总结、卫生发展援助、全球卫生治理和合作伙伴关系试点，预期实现以下对应的四个方面产出：产出 1：提升中国提炼、推广本国在改善健康状况、改进卫生体系等领域经验的能力；产出 2：增进中国政府和学术界对全球卫生发展合作（包括双边和多边）最佳实践的理解；产出 3：提高中国政府和学术界能力，为全球卫生政策的制定和治理做出贡献；产出 4：与发展中国家开展伙伴合作试点，推广中国的卫生经验以及在发展合作方面的最佳实践。项目活动主要在国家和机构两个层面开展，其中国家层面的活动主要在“领域 3 全球卫生治理”下。此外，领域 1 和领域 2 的活动的产出为领域 4 活动的设计和开展提供证据方面的支持。项目设计的结果链详见图 1。

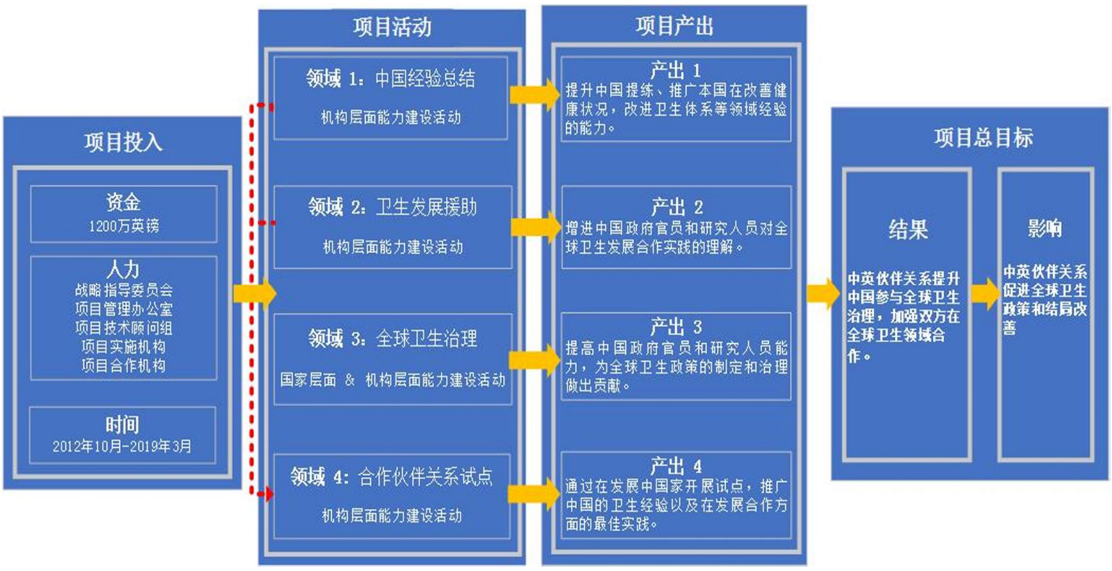

图 1　项目设计结果链示意图

2.1.2 具体活动设计

在国家层面，项目强调战略先行理念，关注平台搭建和机制建立。主要活动包括：开展中国全球卫生战略相关研究；建立中国全球卫生网络；建立中英全球卫生对话机制等。在机构层面，项目坚持采用“干中学”的方式提升能力，强调跨学科、跨部门、跨地域开展全球卫生工作的理念，关注为研究与决策间搭建桥梁。活动的设置主要围绕五类能力的提升：综合研究分析能力、传播与培训能力、政策咨询能力、境外实践能力和伙伴关系建设能力。主要的活动类型包括政策咨询与研究、培训（编写培训教材、组织培训）、国际会议（组织或参与）、发表出版研究成果、长短期国际交流学习活动（国际组织借调、现场观摩、短期考察等）、境外干预试点等。具体的项目活动设计见表 1。

表 1 项目活动设计思路

<table>
<tr><td colspan="6">国家层面</td></tr>
<tr><td colspan="6">关注：战略先行理念、平台搭建、机制建立</td></tr>
<tr><td rowspan="3">主要活动（均在领域 3 下开展）</td><td colspan="5">中国全球卫生战略研究</td></tr>
<tr><td colspan="5">中国全球卫生网络建立</td></tr>
<tr><td colspan="5">中英国家层面全球卫生对话机制</td></tr>
<tr><td colspan="6">机构层面</td></tr>
<tr><td colspan="6">关注：干中学的方法；跨学科、跨部门、跨地域理念；搭建研究与决策间的桥梁</td></tr>
<tr><td>主要活动类型</td><td>综合研究分析能力</td><td>传播与培训能力</td><td>政策咨询能力</td><td>境外实践能力</td><td>伙伴关系建立能力</td></tr>
<tr><td>领域 1:
中国经验总结</td><td>研究</td><td>参与和组织国际会议
发表出版研究成果</td><td>编写政策简报</td><td></td><td>联合研究</td></tr>
<tr><td>领域 2:
卫生发展援助</td><td>研究</td><td>组织培训，编写培训教材
参与和组织国际会议
发表出版研究成果</td><td>提供政策咨询
编写政策简报</td><td>现场观摩
短期考察
参与国际会议或境外培训</td><td>联合研究
联合培训</td></tr>
<tr><td>领域 3:
全球卫生治理</td><td>研究</td><td>组织培训，编写培训教材
参与和组织国际会议
发表出版研究成果</td><td>提供政策咨询
编写政策简报</td><td>借调国际组织
短期考察
参与国际会议</td><td>联合研究
联合培训</td></tr>
<tr><td>领域 4:
合作伙伴试点</td><td>研究</td><td>组织培训
发表出版研究成果</td><td>编写政策简报</td><td>开展境外试点</td><td>开展境外试点</td></tr>
</table>

2.2 项目组织结构

该项目由中国国家卫生健康委员会、中国商务部和英国国际发展部（DFID）的代表组成项目战略指导委员会（Strategic Oversight Committee, SOC，以下均用英文缩写），负责项目的宏观管理，并全面领导项目实施。项目管理办公室（Project Management Office, PMO，以下均用英文缩写）设立在中国国家卫生健康委项目资金监管服务中心内，负责项目的日常运行管理。项目技术顾问组（Technical Advisory Group, TAG）由独立咨询专家和机构（WHO 中国办公室）组成，负责为项目实施活动提供技术建议和指导。项目具体技术活动由众多项目实施机构

（Project Implementing Agencies, PIAs）以及他们的合作机构(Project Cooperative Agencies, PCAs) 和试点地区（Pilot Areas）负责组织实施。项目组织结构如图 2。

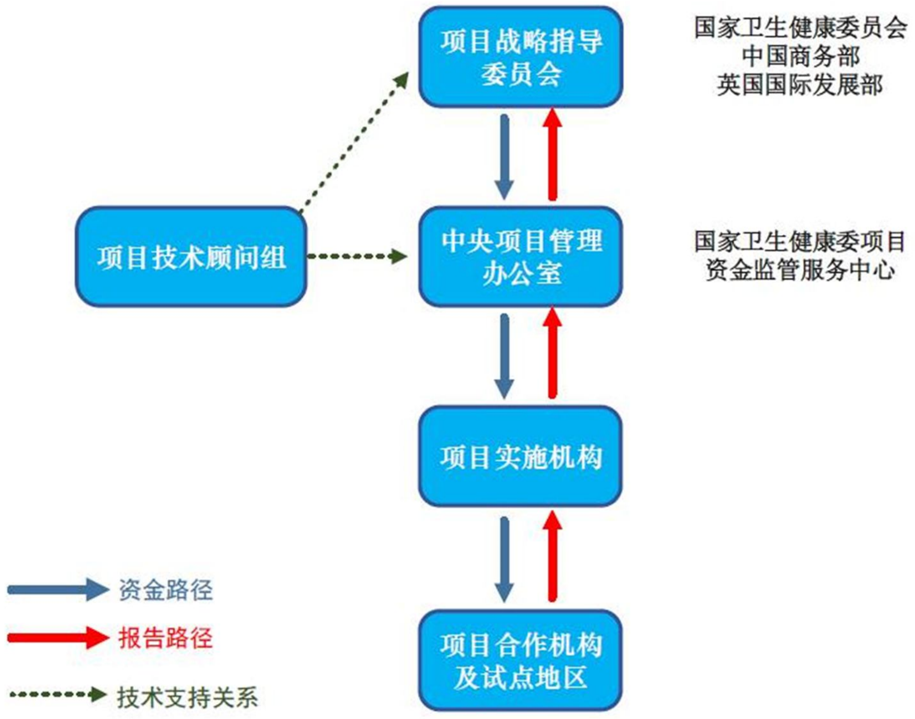

图 2　GHSP 组织结构图

2.3 项目实施

2.3.1 项目管理规则

作为 GHSP 项目的重要文件之一，《项目管理手册》明确了项目的全部管理规则。该手册由 PMO 在项目初期制定，经 SOC 审批后在项目全周期内执行，所有参与项目决策、管理、技术支持和实施的机构/个人均需遵照该手册完成项目相关任务。手册的制定基础包括中英两国相关的法律法规、出资方（DFID）的特殊规则、项目各类法律文件，以及国际项目管理的规范等。手册的内容包括：项目组织结构和职责、日常管理、财务管理、采购管理、监测与评价等。

2.3.2 项目决策机制

项目 SOC 会议为项目决策会议，每年两次，分别在 3-4 月和 10-11 月召开，主要议题包括：年度工作计划审批、项目进度审查、项目重要问题和解决方案讨

论等。在 SOC 会议期外，如有需要 SOC 集体决策的重要事项，由 PMO 提出建议，并根据问题复杂程度，通过增加会议或者是邮件批复的方式进行。

2.3.3 项目参与机构和个人的选择

SOC 成员单位在项目准备期间即由中英双方商定，其他的项目相关机构和个人均是项目启动后依次确定的，具体的确定方式为：

（1）PMO:由 SOC 通过邀请招标的方式确定，DFID 驻华代表处与 PMO 签署项目管理协议；

（2）TAG：由 SOC 成员单位根据一致同意的工作任务书（TOR）的要求分别推荐国际和国内专家，并经集体讨论确定具体人选。TAG 专家的变更也需经 SOC 会议讨论决定。TAG 专家的合同前期由 DFID 驻华代表处、后期由 PMO 与专家签署并管理；

（3）PIAs：每项具体任务的实施机构是通过咨询服务采购的途径来选择确定的。依据任务金额、性质和实际需求的不同，GHSP 的咨询服务采购主要包括三种方式：公开招标、邀请招标和直接确定。公开招标和邀请招标的方式可以在较为广泛的范围内选择有意愿，且有一定工作基础的机构和团队实施项目活动，在项目前期使用较多。直接确定的方式是由 SOC 三家成员单位直接协商确定项目实施机构，在项目后期较多使用。主要适用于以下三种情况的任务（a）金额较小；（b）之前任务的自然延续；（c）属于特殊任务只有唯一的机构有职责或能力可以执行。总体而言，采购的执行主要包括以下步骤：准备工作任务书、发布招标公告、评审标书、与中标方谈判项目合同、签署合同等。其中规定具体任务的工作任务书是由 PMO 和 TAG 专家根据项目设计书和批准的年度工作计划共同拟定、经 SOC 批准后最终确定的。PMO 负责招标的全过程，并对每个实施机构的咨询服务合同的执行情况进行管理。

（4）PCAs 和其他。鉴于全球卫生是一个跨学科、跨部门、跨领域和跨国界的事业，GHSP 致力于促进跨界交流与合作，吸引尽可能多的机构参与其中。为此，对于大型任务，项目鼓励实施机构选择和邀请国内/国际的合作伙伴进行联合投标。确定中标后，项目实施机构会与共同投标的合作机构通过签署合同或协

议的方式进行合作，共同完成项目任务。此外，项目还通过建立中国全球卫生网络的方式，吸纳了更多有意愿参与全球卫生的机构参与到项目活动中来。

2.3.4 项目质量控制

项目质量控制过程包括以下几方面内容：（1）逻辑框架的制定：项目实施监测是项目质量控制的重要手段，明确了预期结果的项目逻辑框架是对项目进展进行监测的基本工具以及对项目过程和结果进行评估的重要基础。GHSP 项目在正式启动前即由设计团队制定了逻辑框架，其中清晰列出了项目的目标、投入、里程碑指标和结果指标，以及一些基本假设。结合项目的实际需求，PMO 和 TAG 专家在项目期内对框架部分内容进行了两次调整，并获得了 SOC 的批准。根据项目监测要求，PMO 每年对逻辑框架指标的数值进行统计更新，以随时确定项目进展是否符合预期。（2）项目年度工作计划审批。每年初由项目办和项目实施机构根据项目设计书、逻辑框架、单个任务的建议书（PMO 和各实施机构签署的咨询服务合同中的技术文件）分别制定，由 SOC 进行审批。批准后的年度工作计划将作为各机构开展活动的依据，也是资金支付的基础。（3）项目过程监督和检查。主要包括 PMO 开展的支付相关审查；SOC 开展的半年度进展报告审查和试点现场督导；DFID 特聘专家开展的年度审查；国家审计署开展的年度审计等。此外，试点的实施机构也会组织试点内的现场督导。审查的重点内容主要包括：项目实施进度、逻辑框架指标的达标性、阶段性产出质量和数量、财务支出合理性和规范性、采购程序合规性等；审查依据主要包括：项目设计书、项目逻辑框架、项目管理手册、项目年度工作计划、PMO 和各实施机构签署的咨询服务合同、各实施机构与合作机构签署的协议等。（4）任务完工验收：每个项目实施机构完成其承担的任务时，PMO 均需对其开展完工验收，包括确认产出的数量和质量、确认采购过程合规性、总决算和经费使用合规性等。项目管理全程遵循了“经费支付与产出数量和质量挂钩”的原则，最大限度确保了每个项目实施机构都能够按照计划提交合格的产出，符合 DFID 提倡的“物有所值”（Money for Value）理念。（5）项目独立评价：项目采用第三方独立评价的方式，对项目实施进展和成果产出情况进行全面评价，包括基线调查、中期评价和终末评价。独立评价团队由 DFID 通过国际招标的方式在项目初期选择确定。独立评价组的各

阶段评价报告为 SOC 确定下一阶段工作重点提供客观、公正的证据支持，也可供中英两国政府今后开展相关工作参考。

3.项目主要成果

历经近 7 的实施活动，GHSP 项目取得了超出预期的结果（GHSP 项目逻辑框架主要产出指标的完成情况详见表 2）。在国家层面，GHSP 支持了 5 届中英高级别全球卫生对话，切实深化了双方的相互理解，中英双方初步构建了新型全球卫生合作伙伴关系；研究团队完成的中国全球卫生战略系列报告，为国家层面出台相关战略性文件提供了有力证据支撑；项目建立了中国全球卫生网络（China Global Health Network, CGHN），并帮助其成长为国内全球卫生领域相关机构和专家研讨、交流和相互学习的平台；在机构层面，项目资助了一系列培训及国际交流活动，产出一大批较高质量的全球卫生中英文研究报告、学术论文以及书籍著作或译著，培养了一批有志、且有能力参与全球卫生事务的中国机构和人员队伍。境外伙伴合作试点工作除了改善当地医疗卫生服务提供、利用能力以及健康结局外，更明显增强了中方机构的境外全球卫生发展援助实践能力。项目实施机构还与境内外相关机构建立起相当数量的合作伙伴关系，这些关系反过来又有力促进了项目目标的实现。

本文相关的项目成果产出数据来源于项目实施期间各 PIAs 和 PMO 提交的项目半年度进展报告，以及项目实施结束后 PMO 编撰的 GHSP 完工报告。

表 2　GHSP 项目逻辑框架产出指标完成情况

产出指标	目标值	指标实现情况
**产出 1.1** 由本项目支持，在提炼中国改善健康成果及加强卫生体系建设方面能力得到增强的中国机构数量和这些机构中参与该工作的员工数	34 位个人+10 个机构	98 位个人+11 个机构
**产出 1.2** 由本项目支持，提炼与中低收入国家相关的、中国改善健康成果及加强卫生体系建设等方面经验的文章数量	200	275（87 研究报告+126 期刊论文+48 政策简报+14 出版书籍）
**产出 1.3** 中国和中低收入国家机构建立的研究伙伴关系的数量	10	26
**产出 1.4** 与中低收入国家研究人员和公共卫生官员开展的研究成果分享活动	10	18
**产出 1.5** 中方机构应用证据和临床的有效性提出政策建议和临床指导方针，以提高卫生部门资源配置效率（NICE）	2	4

产出指标	目标值	指标实现情况
**产出 1.6** 中国机构与中低收入国家分享提高配置效率的经验（NICE）	2	4
**产出 2.1** 结合国际最佳实践，撰写卫生发展合作方面的政策文件或项目文件数量	15	67
**产出 2.2** 开发政策和项目相关研究，从而反映国际卫生发展合作实践（卫生发展合作政策或项目相关的研究论文数）	20	35
**产出 2.3** 建立中国核心机构，发挥其智库作用，能够提供卫生发展合作方面的培训 （在卫生发展合作最佳实践方面，能起到智库作用和提供培训的机构数）	4	6
**产出 2.4** 通过本项目支持，培养中国专家顾问队伍，可以为中国政府、全球卫生相关机构、中低收入国家和/或机构的卫生发展合作提供技术支持（咨询专家数）	50	135
**产出 3.1** 由本项目支持，建立或加强中国全球卫生网络，能够为项目实施机构和其他相关机构提供研讨、发展和相互学习的平台	全球卫生网络建立个发挥作用	全球卫生网络于 2015 年 12 月成立并正常运营
**产出 3.2** 为中国国家全球卫生战略所开发的提案数和完成的政策相关研究数	10 项研究 +1 项提案	11 项研究+1 项提案
**产出 3.3** 通过中英高层全球卫生对话，不断加强中英全球卫生合作	联合开展全球卫生工作	中英全球卫生对话已定期召开 5 届；并于 2018 年在埃塞俄比亚开展非州疾控中心联合考察活动
**产出 4.1** 试点合作伙伴关系的数量	2	4
**产出 4.2** 纳入中国卫生发展合作的经验以及国际卫生发展合作的实践的试点数量	2	4
**产出 4.3** 通过试点合作伙伴关系提高中国参与全球卫生合作的能力	2	4

3.1 国家层面

3.1.1 中英全球卫生对话

自 2013 年起，GHSP 项目累计支持 5 届中英全球卫生对话。中英全球卫生对话在双方相关部门的司局长级官员之间展开，它也是中国与主要西方大国之间的首个全球卫生对话机制。该机制的形成有助于两个全球卫生大国优势互补，形成合力，更好和更加有效地贡献于全球卫生治理，最终改善全球的健康结局，促进可持续发展目标（SDGs）的实现。基于当时的国际国内卫生发展形势、及双

方关切的重点，每次对话的主题均有所不同，涵盖抗生素耐药性、全球卫生安全、世界卫生组织改革、全球卫生治理谈判和全民健康覆盖等多个方面（详见表 3）。

表 3　历届中英全球卫生对话情况一览表

时间	地点	涉及主题
第一届<br>2013 年 3 月 11 日	英国伦敦	全民健康覆盖、 疟疾和脊髓灰质炎防控药物获取、全球卫生政策与治理、后千年发展目标与健康等
第二届<br>2014 年 11 月 22 日	中国上海	基本药物获取、 埃博拉应对、妇幼卫生、全球卫生伙伴关系与治理、 后千年发展目标与健康, 中国全球卫生战略等
第三届<br>2015 年 9 月 14 日	英国伦敦	抗菌药物耐药性与耐药性疟疾、后埃博拉时期卫生合作、可持续发展目标与健康、 世界卫生组织改革等
第四届<br>2017 年 7 月 12 日	中国北京	世界卫生组织改革、 政策更新、在非洲卫生合作、第二轮中英全球卫生合作等
第五届<br>2019 年 1 月 22 日	英国伦敦	新二轮中英全球卫生项目设计、世界卫生组织改革、全民健康覆盖、全球艾滋病、结核和疟疾基金(GFATM), 在非洲全球卫生安全合作等

3.1.2 中国全球卫生战略系列研究

GHSP 项目完成了一整套有关全球卫生战略的研究，共产出 12 份研究报告和 1 份拟定的中国全球卫生战略，为中国政府制定本国全球卫生战略文件提供了翔实的数据、事实和具有建设性的政策建议。研究报告的内容涵盖了“传染病与被忽视的热带病”、“慢性病”、“卫生体系”、“卫生外交”、“环境与卫生”等全球卫生热点领域（详见图 3）。

项目产出中关于中国全球卫生战略的建议，与 2016 年中国中央政府发布的两个文件—《“健康中国 2030”规划纲要》和《“十三五”卫生与健康规划》中的精神高度吻合。《“健康中国 2030”规划纲要》中包括“实施中国全球卫生战略”、“充分利用国家高层战略对话机制，将卫生纳入大国外交议程”和“积极参与全球卫生治理，提升健康领域国际影响力和制度性话语权”等表述[31]；《“十三五”卫生与健康规划》中包括“制订中国全球卫生战略”、“提升我国在全球卫生外交中的影响力和国际话语权”、“继续加强卫生援外工作”和“推进全球卫生人才培养和队伍建设”等表述[32]。这种吻合在一定程度上反映出项目相关研究工作获得了国家卫生健康委员会乃至中国政府最高决策层面关注和肯定。

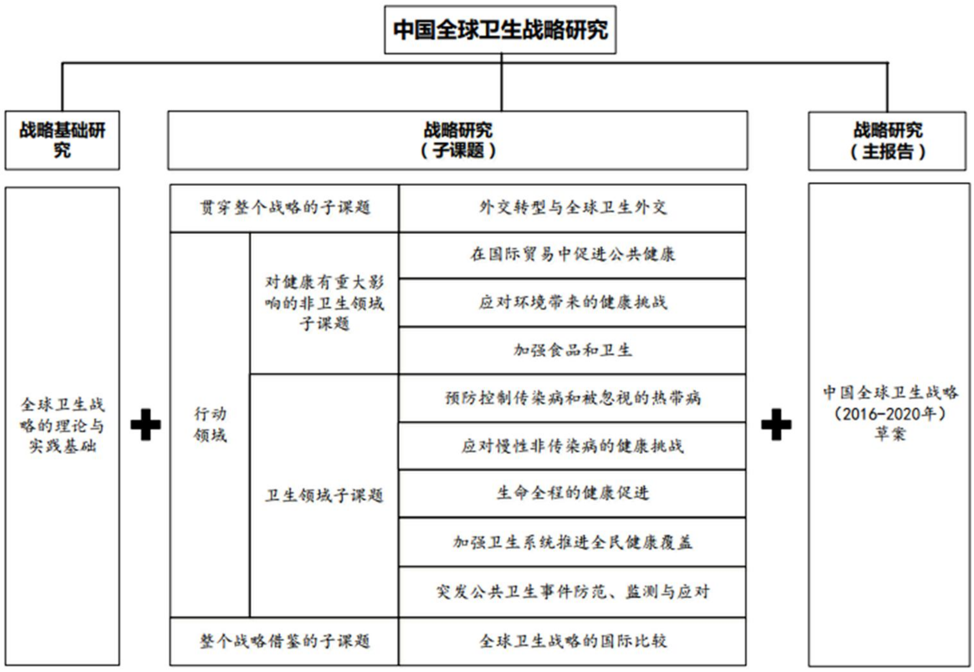

图 3　中国全球卫生战略系列研究结构示意图

3.1.3 中国全球卫生网络成立并正常运行

在 GHSP 支持下，中国全球卫生网络（China Global Health Network, CGHN）于 2015 年 12 月 6 日在北京正式成立，北京大学公共卫生学院成为网络首届理事长单位并承担秘书处职能。CGHN 秉持开放包容的原则，鼓励并广泛接纳多种行为体成为网络成员并参与网络活动。截至 2019 年 3 月，中国全球卫生网络成员单位数量已由成立之初的 46 家增至 77 家，分布于中国的 20 个省或直辖市，其中不仅有大学、学术机构和智库，也包括了政府部门及公共卫生机构，以及企业和民间社会组织等（如图 4）。中国全球卫生网络对扩大和巩固项目影响力、持续促进中国的全球卫生事业发挥着重要作用。成立以来，该网络通过召开国际会议、开展能力建设培训、受卫生部门委托开展全球卫生合作的相关研究、与国际机构建立伙伴关系并联合开展调研等方式，为成员机构搭建了一个专业化的全球卫生交流平台，并成为中国全球卫生学界与外部世界沟通互动的一个窗口。

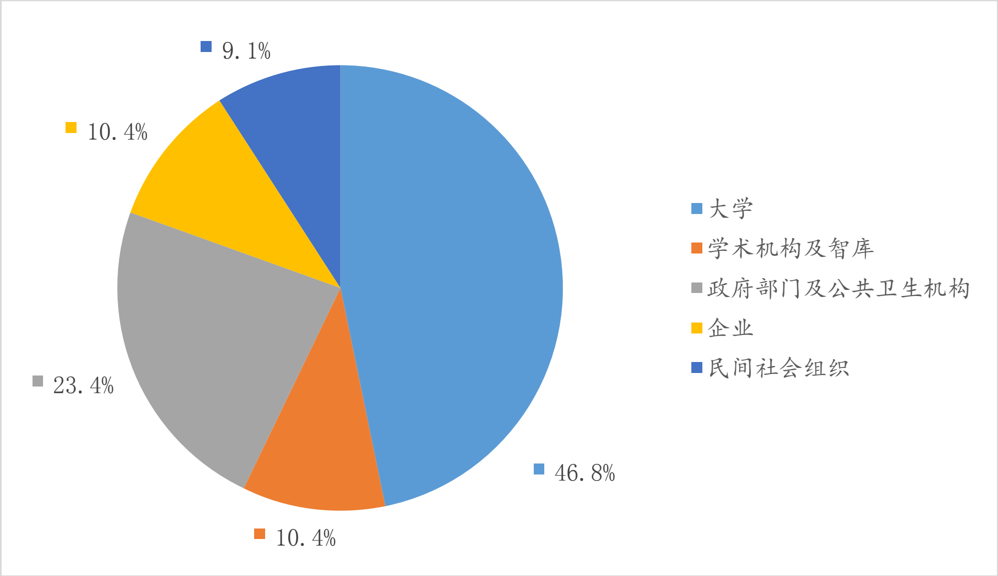

图 4　CGHN 成员性质分布

3.2 机构层面

提高中国机构参与全球卫生工作的能力是项目非常重要的目标。GHSP 项目期内，共有 53 家中国机构以项目实施机构或者项目合作机构的身份参与了项目的活动，它们在贡献于项目产出的同时也在项目实施过程中成长。GHSP 项目支持带动了一批高等院校和研究机构成长为中国全球卫生研究与实践的中坚力量，比如项目中的 6 家全球卫生核心机构：北京大学公共卫生学院，复旦大学公共卫生学院和全球健康研究所、中国疾病预防控制中心所属寄生虫病预防控制所和全球公共卫生中心，以及国家卫生健康委所属的卫生发展研究中心。

3.2.1　政策研究与分析能力

项目实施机构在中国经验总结和提炼、全球卫生发展合作、全球卫生政策与治理以及合作伙伴试点等领域开展了一系列政策研究。通过 GHSP 项目，各实施机构全球卫生研究及分析能力得到实质性提升，产出已陆续通过国内外出版社、学术期刊等以研究报告、论文和专著等形式出版（图 5 显示了不同领域、不同类型产出的数量）。截至 2019 年 3 月项目结束，共计产出 87 份研究报告，126 篇中英文学术论文（英文 57 篇，中文 69 篇）和 14 本书籍著作（含参与撰写章节和译著）。

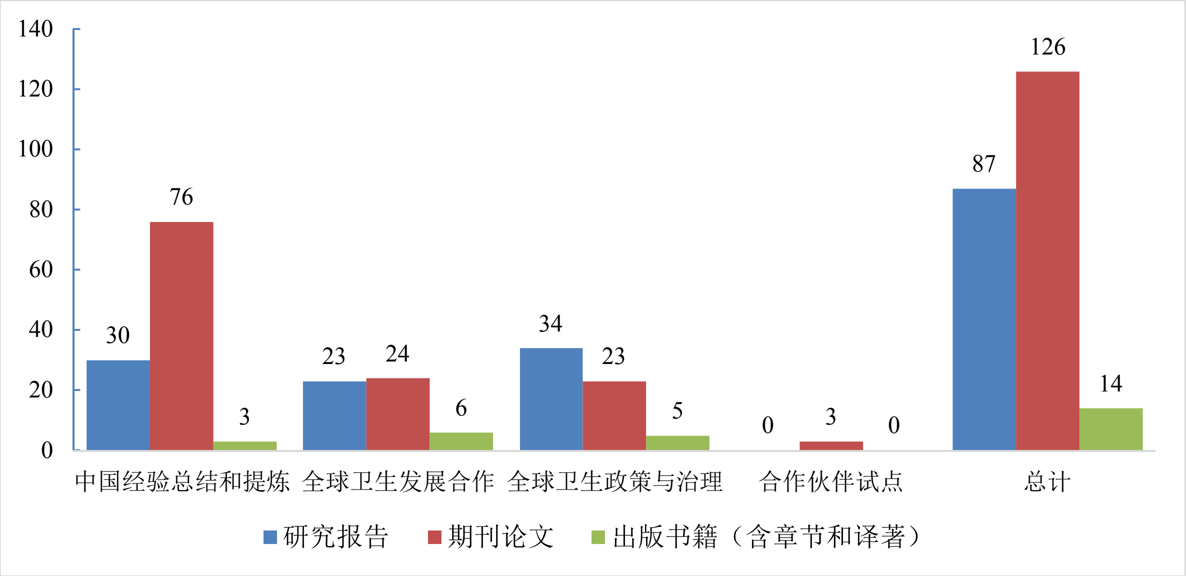

图 5　GHSP 不同领域学术产出统计

3.2.2 传播与培训能力

GHSP 项目通过多种方式提升中国机构的传播和培训能力，如支持实施机构开发卫生发展援助和全球卫生治理的培训教材，组织短期培训课程，参与和开展国际交流活动，支持中国的全球健康专业英文期刊传播等。项目支持相关国际交流活动 109 项，涉及 349 人次。这些活动包括：参加国际会议介绍中国经验，派往发展中国家提供咨询服务，派往国际知名研究机构或高等院校进行短期研修等。项目支持实施机构策划组织主题不同、形式多样的全球卫生培训及实践活动 27 场，培训 1020 人次。参训人员包括政府官员、高等院校和研究机构人员、专业技术人员和国际合作项目管理人员等。GHSP 项目还在相当大的程度上推动了中国高等院校的全球卫生学科建设和人才培养，比如：北京大学利用项目开发的教材开设了《全球卫生概述》和《全球卫生治理》两门研究生课程；复旦大学开发了《全球卫生导论》课程，并已在中国高等院校的慕课（MOOC）平台上线；武汉大学全球健康中心主办的英文学术期刊 *Global Health Research and Policy* 也通过与 GHSP 项目的合作促进了 GHSP 项目部分产出的国际传播。

3.2.3　政策咨询能力

政策简报是把研究成果转化为政策建议、服务于决策的短小而快捷的产出形式。在 GHSP 的重点支持和指导下，项目实施机构依据政策性研究的成果，为中国政府及发展中国家提供了 48 期有关全球卫生重要议题和趋势的政策简报（如

图 6）。若干政策简报得到中国社会科学院信息情报研究院的肯定，被改编成为内参信息直接报送中国政府最高决策层。

项目期间实施机构以不同的形式为中国政府和世界卫生组织提供了专家咨询服务，包括：参与咨询、起草若干全球卫生规划或方案，如“中国全球卫生战略”、“健康亚太 2020 战略规划”、中国卫生健康部门推进“一带一路”卫生交流合作的实施方案（2015-2017）和（2018-2020）、“中国援建 100 所医疗卫生机构方案”、“发展中国家培养卫生人才方案”、“健康中国 2030”规划纲要涉及国际交流与合作的内容等；作为顾问团成员，为国家卫生健康委代表团提供出席 WHO 执委会会议和世界卫生大会的咨询意见；在 WHO 等国际组织的专家委员会中任职，提供疟疾防控、热带病防控以及妇幼卫生等领域的技术咨询意见等。

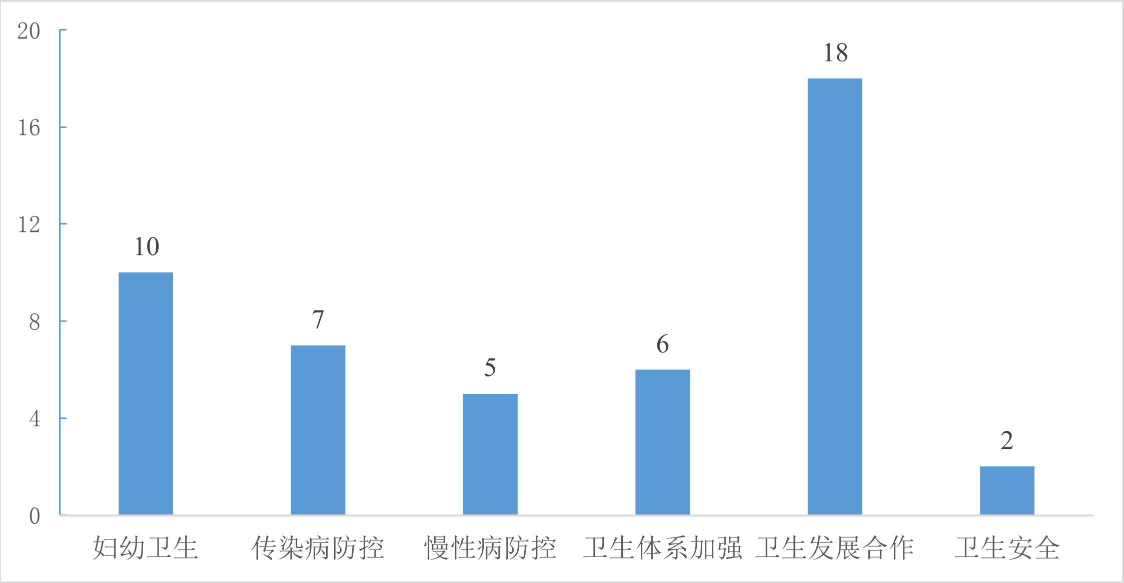

图 6　GHSP 政策简报产出的主题情况

3.2.4 境外实践能力

GHSP 支持实施机构在缅甸和埃塞俄比亚开展妇幼与生殖健康干预试点，在坦桑尼亚开展疟疾防控试点，并开展了其他相关能力建设活动，如：借调若干全球卫生工作骨干赴世界卫生组织等国际机构工作；援助塞拉利昂提高公共卫生能力；开展在非洲工作的国际公共卫生合作伙伴的咨询与研讨，等等。这些活动在以下三个方面提高了中国机构境外实践的能力：

（1）在发展中国家应用中国经验的能力。试点（详见案例 1 和案例 2）工作在设计阶段和实施过程中均根据当地实际对中国经验进行了调整，提高其适用

性，最终实现了试点预期目标。例如，疟疾试点项目中，通过分享中国消除疟疾“1-3-7”工作理念，创新性建立了适宜坦桑尼亚当地的基于社区的快速降低中高度流行区的疟疾负担的干预模式（1,7-mRCT），明显降低了干预社区的疟疾负担，疟疾感染率下降了 70％以上。妇幼卫生试点采用了中国在促进住院分娩中使用的三环策略（链接服务需方、支付方、供方），但根据各地的情况，选择了不同的人员作为链接者，比如中国选择的是农村家庭接生员，而埃塞选择的是当地的卫生拓展人员，缅甸是当地的助产士助理。通过他们的工作，明显改善了试点地区的孕产妇保健、新生儿保健和避孕节育服务的提供和使用状况，如埃塞项目地区住院分娩率从 28%提高到 55%；缅甸项目地区从 30%提高到 53%。

（2）管理卫生发展合作项目的能力。试点国家与中国政治经济制度、社会结构、语言文化的差异较大，且试点工作时间较长、金额较大，涉及众多利益相关方，这些都为项目管理带来巨大挑战。中方实施机构在与当地合作伙伴开展工作的过程中，不断从实践中学习，积累经验，逐步提升了项目管理的意识和专业技能。同时，在面对中国某些现行体制机制与“走出去”的国家政策不匹配的现状时，中方人员也历练了其发现问题，主动寻求解决方案的能力，使得最终试点目标得以实现。

（3）与国际社会合作开展卫生发展合作的能力。中国机构在项目实施过程中获得了近距离观察和学习“国际社会如何在卫生发展和全球卫生治理方面开展工作”的机会，如项目支持中国卫生健康委官员参与英国和世界卫生组织在非洲的联合考察；支持 7 名全球卫生工作骨干借调到世界卫生组织、全球基金等机构工作。在境外实践过程中，中国机构逐渐认识到与国际社会各方保持紧密联系的重要性，培养锻炼了与外界沟通的能力。例如，在塞拉利昂的中方公共卫生团队与当地政府、来自英美国家的合作伙伴以及在当地的国际组织建立了技术沟通机制，及时分享工作成果。

案例 1：坦桑尼亚疟疾控制海外试点项目
I. 实施机构：中国疾病预防控制中心寄生虫病预防控制所（NIPD）
II. 合作伙伴：坦桑尼亚依法卡拉卫生研究所（IHI）、坦桑尼亚国家疟疾控制项目署（NMCP）、 坦桑尼亚国家医学研究所（NIMR）
III. 试点目标:
总目标：应用中国疟疾防控经验结合世界卫生组织 T3 策略开展试点，将试点社区的疟疾负担较项目开始时降低 30%以上
具体目标：（1）通过提高病例病原学检测率（T1）、病例规范治疗率（T2）和追踪管理（T3），增强试点社区的疟疾防治与控制能力；（2）通过培训当地卫生人员有效利用现有设施和提高社区和合作伙伴参与疟疾

控制的能力，优化原虫和媒介的监测响应系统并提高信息报告的能力；（3）评估试点社区应用中国经验结合 WHO T3 策略的实施效果及成本效益；（4）通过总结试点项目经验与教训，为坦桑尼亚国家疟疾防控项目和中国政府今后开展发展援助工作提供政策建议。

IV. 实施周期与地区:实施周期为 2015 年 5 月至 2017 年 6 月 30 日；实施地点位于坦桑尼亚南部的鲁菲季 (Rufiji) 地区。

V. 主要活动:

（1）在试点社区建立流动镜检站并引入电子报告系统以改善疟疾病例的诊断、治疗和疫情报告，以迅速减少高危人群的疟疾负担；（2）通过中方专家与当地专家在实施社区共同实施项目，分享中国经验并提供现场技术支持；（3）通过培训试点社区的临床医生，卫生服务人员和试点地区志愿者的疟疾病例管理技能，提高当地疟疾防控能力；（4）通过基线调查、中期评估和末期评估，评估基于社区防控模式的成本效益；（5）通过总结试点项目经验与教训，为中国政府的对外援助模式和坦桑尼亚政府未来疟疾控制和消除的战略规划提出建议。

VI. 主要产出:

（1）通过实施 76 轮基于社区的快速筛查和治疗（1,7-mRCT），干预社区疟疾感染率下降了 70％以上，超出了长效蚊帐的干预效果（LLINs)；（2）通过分享中国消除疟疾“1-3-7”工作理念，创新性建立了适宜当地的基于社区的快速降低中高度流行区的疟疾负担的干预模式（1,7-mRCT），并在非洲共同实施了三方合作项目；（3）通过各方技术人员、政策制定者和实施者分享疟疾控制和消除的经验、技术和产品，共同在非洲搭建了政策对话和转化平台；（4）通过加强基于社区的能力建设和当地卫生系统、实施基于社区的快速筛查和治疗策略（1,7-mRCT）、分享中国丰富的疟疾控制和消除经验等，将影响中国对外援助模式，支持中国成为重要合作伙伴之一，加快非洲国家疟疾控制和消除进程。

案例 2： 缅甸及埃塞俄比亚妇幼卫生海外试点项目

I. 实施机构：复旦大学全球健康研究所

II. 合作伙伴：英国玛丽斯特普国际组织（MSI）、玛丽斯特普缅甸（MSIM）、玛丽斯特普埃塞俄比亚（MSIE）、昆明医科大学健康科学研究所、提露内丝- 北京中埃友谊医院、埃塞俄比亚中国医疗队

III. 试点目标:

（1）提升中方机构在低收入国家参与全球卫生发展合作相关活动，开展相关研究及健康干预项目的专业能力，为支持中国全球卫生发展规划提供可靠证据；（2）通过应用基于循证方法确定的中国成功经验，在缅甸和埃塞俄比亚的试点地区开展生殖健康、孕产妇、新生儿和儿童保健领域的干预活动，以提高当地妇女儿童对相应健康服务的利用，从而改善其健康水平；（3）通过试点实践，探索中国在其他发展中国家实施卫生发展合作项目的经验、教训以及有效模式，特别是尝试与国际非政府组织进行多方合作，以及与中国援外医疗队和中埃友谊医院联合开展培训项目的可行做法。

IV. 实施周期与地区:

埃塞俄比亚试点地区实施周期为 2015 年 11 月至 2017 年 6 月，覆盖人口约 25 万人；缅甸试点地区实施周期为 2016 年 10 月至 2018 年 6 月，覆盖人口约 30 万人。

V. 主要活动:

（1）通过人员外派和现场督导提高中方机构在低收入国家工作的能力，通过培训项目和“干中学”实践增强其专业能力；（2）应用中国促进妇幼与生殖健康的成功经验和方法，结合试点国家的国情现状，研究制定符合当地实际的试点干预实施方案；（3）通过在当地开展社区动员、加强社区和卫生机构之间的链接、减少影响卫生服务可及性的经济和地理障碍、以及提升卫生服务质量等一系列措施，改善两国试点地区的孕产妇保健、新生儿保健和避孕节育的服务提供，提高相关人群的服务利用意识；（4）开展三轮师资培训项目，在埃塞尔比亚的提露内丝-北京中埃友谊医院建立高级助产培训中心，购置必要的培训设备，提供相关专业培训。

VI. 主要产出：

（1）**干预有效性：埃塞试点地区**四次及以上产前检查率由项目开始时的 5%上升至项目结束时的 47%（全国水平为 27%）；住院分娩率由 28%增至 55%（全国水平为<21%）。**缅甸试点地区**四次及以上产前检查率和产后检查率分别增长了 19%和 10%；住院分娩率由项目开始时的 30%上升至结束时的 53%。

（2）**伙伴关系建立方面：**中方高校与国际非政府组织、埃塞俄比亚和缅甸当地政府、中国援外医疗队以及中埃友谊医院紧密合作开展该试点项目，在埃塞俄比亚成功建立一个高级助产培训中心，复旦大学为埃塞俄比亚累计培训 13 名助产士；
（3）**中方团队能力提升方面：**试点项目实施期间 46 位中方人员累计在两国试点地区工作 885 人日，共组织举办 17 次培训会、7 场联合讲座和 5 场个人咨询会议，累计提供培训达 320 人次。

3.2.5 建立合作伙伴关系的能力

项目实施过程中，6 家全球卫生核心机构与数家国内外合作方建立起稳定的伙伴关系（如图 7）。国内合作伙伴涵盖卫生部门、高等院校、科研机构、医院和部分医药科技公司；国际合作伙伴包括世界卫生组织、玛丽斯特普国际（MSI）、健康扶贫行动（HPA）等国际性非营利机构，以及 20 多个发达国家和发展中国家的高等院校、专业研究机构和其他民间社会组织。GHSP 项目期内，项目实施机构通过借鉴学习，取长补短，加强了自身能力；同时与合作伙伴在沟通中磨合，探索合作共赢之道。工作过程中，实施机构除在学术方面大受裨益外，更是在实践中不断学习如何合作、如何管理才能使跨国、跨文化、跨学科的团队达到共同目标，这些经验促进了建立于各个层面上的合作伙伴关系的健康发展。此外，在亚非发展中国家的试点工作也增进了中国、英国和试点国家政府及其他利益相关方的相互了解，为今后进一步开展跨国卫生合作奠定了基础。

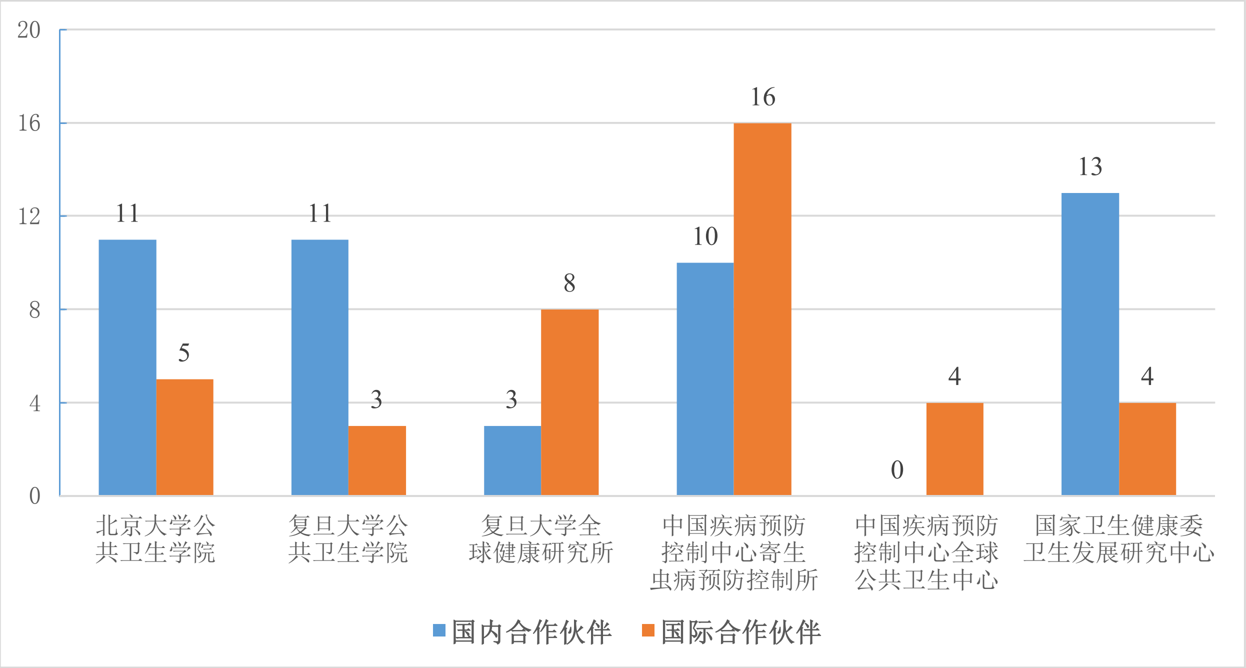

图 7 GHSP 全球卫生核心机构国内外伙伴关系

4. 讨论

4.1 严谨的项目设计是成功的前提

GHSP 项目在 DFID 历年的年度审查中始终获得 A 或 A+的评级。2018 年度审查报告中指出：项目在物有所值方面超出了项目设计书中的期待。项目之所以能取得这样的成就，首先是与项目严谨合理的设计密不可分的。

结构合理的设计团队是项目设计的关键。项目的设计历时一年多,设计团队由英国、中国和非洲三方专家组成，分别对国际发展援助和英国发展合作的政策和做法、对中国更好地参与全球卫生的现状和面临的挑战、以及对非洲在卫生发展领域的需求有深入和全面的了解。他们的组合为 GHSP 项目的设计提供了所需的专长，最大限度地确保了设计的严谨合理。为使中国通过项目提高的能力能更切合发展中国家的需要，除了咨询国内有关管理人员和专家以外，项目设计团队还专门前往非洲实地考察了解情况，走访了乌干达政府部门和高层领导，参观了该国的专业机构，听取了当地的主要国际合作伙伴的意见，还参观了中国既往的卫生援助项目现场，比如援建的医院、当地医疗队等。

找准中国全球卫生能力的不足是项目设计的基础。设计团队经过分析，认为中国在建设卫生系统和促进人民健康方面的成就获得国际公认，中国学界对中国卫生发展的经验也不乏研究分析，但是这些研究均欠缺外部适用性的视角，在全球知识库中也没有得到充分体现；中国长期对发展中国家提供卫生援助，但援助的主要形式是派遣医疗队和建设卫生基础设施，对全球卫生领域其它发展援助方的做法，特别是当代国际卫生发展援助的最佳实践缺乏了解[33, 34]。因此，中国亟需借鉴国际发展援助的成熟经验，提高针对公众健康问题和重大疾病开展公共卫生干预，以及与其它发展伙伴协调行动的能力；随着中国综合国力的不断提升，中国希望积极参与全球卫生治理，尤其是要在相关国际标准、规范、指南等的研究、谈判与制定过程中增强话语权，为全球卫生贡献更多的中国智慧与中国方案。但是，中国缺乏参与全球卫生治理和政策制定的能力。上述三方面能力不足的问题，成为设计团队提出 GHSP 项目预期产出的基础。

项目产出之间逻辑联系是项目整体性的保证。根据前述中国参与全球卫生三方面能力的分析，项目提出了对应的三个产出，即提高提炼、传播、应用中国在

改进健康产出和加强卫生系统方面经验的能力（产出 1）、增进中国官员和研究人员对国际健康发展合作（包括双边和多边）最佳实践的理解（产出 2）、以及提高中国官员和研究人员能力，使其为全球健康政策制定和治理方面做出贡献能力（产出 3）。能力提高最终必须体现在参与全球卫生的实践中，为此，本项目将伙伴合作试点作为产出 4，即通过中英和发展中国家的三方伙伴关系的试点项目，把产出 1（研究总结的中国经验）和产出 2（学习到的国际发展最佳实践）结合起来，应用到选定的 1-2 个非洲和亚洲国家中，为改善人群健康和加强卫生体系做出贡献。这既是提高能力的最终目的，也是对能力提高的检验。项目还期望产出 3 所提高的国家能力为试点项目提供政策支持。这样的逻辑关系将项目不同部分连接成一个整体，使项目结果能够产生真正的影响。

4.2 以务实合作建立可持续发展伙伴关系

联合国 2030 年可持续发展议程把“加强执行手段，重振可持续发展全球伙伴关系”作为一个重要目标，指出：“如果不加强全球伙伴关系并恢复它的活力，就无法实现可持续发展议程的宏大目标和具体目标”，“加强国际社会对在发展中国家开展高效的、有针对性的能力建设活动的支持力度，以支持各国落实各项可持续发展目标的国家计划，包括通过开展南北合作、南南合作和三方合作”是可持续发展能力建设的具体目标[35]。GHSP 项目在这方面提供了一个很好的案例，具体体现在两个方面：

一是探索将“南北援助”转变为“南北合作”的具体路径：传统的南北援助（“援助方-受援方”模式）的核心是北方国家出资帮助南方国家发展。GHSP 项目下的中国和英国虽然也属于南方国家和北方国家的关系，但是这种关系已经由“援助”成功转变为了“合作”。GHSP 的目标被设立为“建立中英卫生新型合作伙伴关系，加强双方在全球卫生领域的合作，提升中国相关能力，共同促进全球卫生状况的改善”，体现了目的和方式两方面的变化：（1）“以促进受援国自身发展”为目的转化为以“加强受援国对全球健康做贡献”为目的；（2）“以单纯的资金援助为合作方式”转化为以“双方建立长远的合作伙伴关系”。

具体而言，GHSP 中合作伙伴关系建立在两个层面上：（1）国家一级的政策对话。GHSP 项目建立了中英双方高层定期沟通机制，即中英全球卫生对话，便于两国政府就全球卫生热点问题交换意见，促进双方的相互理解，为两国在全球

卫生领域就重大卫生健康议题寻求共识，在全球卫生治理中开展协作提供了一个定期交流的平台。（2）机构一级的技术合作：项目鼓励中英双方机构和人员开展各类联合行动，如：在亚非发展中国家的试点、卫生发展援助旗舰培训、全球卫生政策研究、跨文化环境下的项目管理等。除了两国政府部门的密切接触外，中方实施机构与英方的 10 余所机构，包括科学研究机构、智库群体、民间社会组织等建立了联系，这种联系在项目结束时已发展成为比较紧密的和具有实质性内容的合作伙伴关系，且经由这些合作伙伴关系而诞生的产出已经成为公共产品，贡献给国际社会和其他发展中国家。

这种南北合作超越了一般的务虚性、意向性沟通和原则性共识，而是脚踏实地，以精心设计的具体项目为抓手，通过在国家间开展定期对话，在机构间开展联合活动，跨越障碍，从陌生到熟悉，从质疑到理解，逐步形成了双方都能接受的共同协作工作模式，并使得这种合作关系巩固提升，具备可持续的动力和可能性。经由 GHSP 建立的这种南北合作伙伴关系可以成为一个样本，为英国和其他新兴国家的合作，以及中国与其他西方国家在卫生健康乃至更广泛的发展领域的合作提供借鉴。

二是探索三方合作的开展模式：发展援助领域的三方合作，一般指一个提供资金并拥有传统发展援助经验的发达国家（或国际机构）与另外一个具备一定知识与能力的发展中国家携手（如中国、印度），面向另一个或一批接受发展援助的发展中国家开展合作[36]。越来越多的利益相关方认为，这种三方合作是连接南南合作与南北援助的有益渠道，是实现有效发展合作的一种方式。中国与国际组织既往开展过非卫生领域的三方合作，取得了一些成效。但是中国与西方发达国家携手开展卫生健康领域的三方合作案例极少，GHSP 正是在这方面开展了积极的探索。

GHSP 项目一共开展了三个试点：分别是坦桑尼亚的疟疾防控试点；埃塞尔比亚的妇幼卫生试点；缅甸的妇幼卫生试点。具体的操作方式为：英方提供资金，中方、英方和试点国家合作机构共同确定试点主题，中方实施机构和试点国家合作机构负责试点方案设计和具体实施（其中妇幼卫生项目试点国的合作机构是英国注册的民间社会组 MSI），英方根据需要提供必要的技术和管理方面的指导。在合作中，英方提供的资金和其对发展中国家援助工作的管理经验、中方提供的

自身发展经验和在妇幼和疾病防控领域的技术和技能以及试点国合作机构的意愿和努力，三者的有机结合使试点获得了成功。

但是合作中也有一些问题，值得探讨，比如，三方合作涉及的利益相关方较多，而各方的立场和关注点有所不同，因此项目前期花费的时间和沟通成本非常大，项目实施期问题解决的过程也比较漫长；此外，虽然项目遵循尊重试点国意愿的原则，但是由于三方合作的发起者是中方和英方，且试点是由中方机构与试点国的合作机构（非政府部门）协商确定的，所以过程中试点国政府参与度不高。事实上，当地政府对试点的了解和认可恰恰是试点结果获得可持续推广的先决条件。虽然项目在后期采取了一些行动来弥补这个缺陷，但是如果三方合作一开始就能够从政府层面开始沟通，且在合作中有具体机制确保政府的深入参与，将会大大提高合作的可推广性和可持续性。

4.3 深化全球卫生参与尚需多方面改革

GHSP 项目启动之初，中国的全球卫生事业，特别是相关的知识积累、研究和教学，都处于起步阶段。卫生健康系统及其他部门中，鲜有决策者和专业人员对当代全球卫生议题给予持续关注，更遑论对此有较为全面深刻的认识与分析。GHSP 的实施使得先进的全球卫生理念及相关学说在中国的卫生相关部门得到了比较广泛的传播，项目支持的政策性研究，特别是战略性研究，为中国政府更加积极地参与全球卫生治理、谋划和改善卫生发展援助提供了信息支持。此外，项目突破了传统的中国对外卫生发展援助做法，在三个亚非发展中国家开展卫生健康干预试点，探索中国开展卫生发展合作的新方法、新模式，对如何在发展中国家实施公共卫生干预项目，如何通过三方合作提高卫生发展援助有效性，如何与存在于当地的各种民间社会组织（包括国际非政府组织）协调等问题进行了有益尝试和探索，为中国下一步卫生发展援助提供有益借鉴。然而，项目实施的过程也并不是一帆风顺的，在发现问题和解决问题的过程中，项目管理和实施机构对影响中国全球卫生参与的瓶颈问题和未来的努力方向有了更深刻的认知。

一是新型的卫生发展援助亟需体制机制和其他配套措施支持。中国政府近年在全球卫生领域做出了很多有影响力的承诺，比如与“一带一路”沿线国家开展卫生健康合作，援建非洲疾控中心，实施中非新发再发传染病、血吸虫、艾滋病、

疟疾的疾控合作项目等[37,38]。要想顺利地落实这些承诺，创新卫生发展援助势在必行。我们认为：中方机构和人员要想顺利地走出国门，开展新型卫生发展援助工作，除了要解决自身经验和技术优势在发展中国家落地的可行性及适应性问题外，更需要同步解决三方面问题：（1）解决中国"走出去"的体制机制和配套操作规范。比如，试点实践中发现：中国现行外汇管理、公立机构人员出访规定、出入境管理等方面的限制，以及出境人员身份和待遇、人员健康和人身安全保障的规定已经无法满足境外执行长期项目的实际需求，造成了进程的延缓、管理成本的增加，人员的安全风险升级等。这些问题单靠实施机构自身的努力是无法解决的，需要国家层面的统筹安排。（2）解决谁"走出去"的问题。GHSP 试点的实施机构主要是高等院校和公共卫生机构，他们的特点是技术上有很强的优势，但是管理能力相对较弱。随着"走出去"需求的增加，除了这些机构，中国也需要参照国际社会既往的实践，有意识地培育一些国际化水平较高、具备可持续"走出去"能力的民间社会组织参与这方面的工作。（3）解决"走出去"人员的项目管理意识和跨文化管理技能的问题。GHSP 试点虽然最终均取得了预期效果，但是过程曲折，特别是早期进展缓慢，其中的一部分原因是人员缺乏专业管理技能，缺乏对国际通行项目管理规则和跨境项目管理风险的认知。GHSP 项目执行过程中反映出中国相关机构普遍缺乏项目管理能力，而它是确保卫生发展援助资源（中国纳税人的资金）被合理和有效使用的基本能力，对未来的新型卫生发展援助的工作开展中至关重要，需要有计划性地逐步培养，并有意识地通过三方合作，向传统的援助国或国际组织学习。

二是全球卫生需要多部门参与治理。国际社会特别是英国等发达国家的经验表明，全球卫生是需要不同政府部门和社会各界各利益相关方共同协调和促进的综合性事业，绝非卫生管理部门一家的责任。GHSP 项目虽然在全球卫生理念普及和意识提高方面发挥了重要作用，但主要还是集中在卫生健康部门。针对其他政府部门（如外交部、财政部、海关总署等）的倡导做得非常有限。国家于 2016 年发布的《健康中国 2030 规划纲要》中明确提出"将健康融入所有政策"[31]。2019 年 7 月发布的《健康中国行动（2019-2030）》又明确了各部门在行动中的具体职责，这些都为中国多部门参与全球卫生治理奠定了基础[39]。中国尚需就全球卫生议题更多地开展各政府部门之间的信息共享、磋商和协调，并待时机成熟时制

定和发布全政府的全球卫生战略，只有这样前述所提的中国“走出去”的体制机制和其他配套措施的问题才能得以解决。2018 年中国政府设立了国家国际发展合作署，这无疑有助于把卫生健康议题融入发展援助，也为日后在国家层面统筹制定全球卫生战略、建立相关体制机制，出台配套政策提供了可能。

4.4 全球卫生研究和政策咨询工作尚有很大改善空间

全球卫生相关政策的制定需要大量优质证据的支持，因此提高研究者的分析能力和提供高质量政策咨询的能力，搭建研究与决策间的桥梁正是 GHSP 项目研究活动设计时关注的重点。一方面项目鼓励研究者和决策者的紧密结合，最典型的案例就是“中国全球卫生战略研究”，这是项目的一项重要的国家级活动。该研究小组从设计期就邀请有关政府主管部门参与，每一个阶段性成果也都请主管部门参与评审。在项目期间，研究小组被政府主管部门确定为“全球卫生战略”政策文件的起草团队，直接接受主管部门的指导。这种研究者与决策者的紧密结合促进了研究者对政府需求的了解以及研究成果被政府部门的认可，对研究成果向政策的转化起到催化作用。实际上，研究活动结束不久，主管部门的“中国全球卫生战略”也作为部门政策文件出台了。另一方面项目对所有研究团队提供了有关政策简报撰写的培训和指导，主要研究成果除了陆续通过国内外出版社、学术期刊发布外，也同时以中英文政策简报的方式呈现，为决策者提供高质量的经过浓缩加工的情报信息，使其可以在最短的时间内准确了解该议题的概况，引起相应的关注。

虽然 GHSP 支持了上述工作，但是项目过程中也发现一些问题，现就全球卫生研究与咨询提出以下三点建议，供今后工作参考：

一是在循证决策的互动中，研究者和决策者双方是同等重要的。项目在这个过程中只重点关注了“改进研究者提供信息和政策咨询方面的能力”，但是未能在“决策者有选择地接收和使用信息”方面开展特别的工作，而实际上在推动研究成果转化为政府政策的过程中后者显然更为重要。这是今后类似的项目可以考虑支持的方面。

二是在研究者与决策者紧密结合的过程中，研究者需要意识到，这种结合在带来益处的同时也会带来挑战。比如研究者需要在尊重体制内官员的意见和保持

研究小组的独立性间保持平衡；需要在科学研究的“理想性”与决策者思考问题的“现实性”间保持平衡。这对研究者的政治智慧是一个非常大的考验。

三是全球卫生相关研究在深度和广度方面均有待拓展。GHSP 项目针对不同主题开展了大量研究，但是其中有两个主题的研究开展得不足够，亟待在未来工作中加强。它们分别是（1）中国卫生经验的国际适应性。虽然项目突破性地尝试从外部视角开展对中国卫生体系建设、妇幼卫生保健、疾病防控等方面经验的国际适应性方面的研究，但是由于这类研究本身具有方法学和实施方面的难度，最终并未能取得预期数量的高质量的研究结果。事实上，中方对中国经验在国际背景下的适应程度的准确把握，将成为创新中国卫生发展援助的一个前提。中国今后的卫生发展合作，特别是同“一带一路”沿线国家的合作，对于这方面的研究和知识将有大量需求。（2）中国利用既往国际卫生发展援助资金的经验和教训。GHSP 项目重点研究和分析了“中国既往为其他发展中国家提供卫生援助”的情况，但是对“中国既往如何有效利用传统援助国和国际组织援助资金”的研究和分析极少且不够深入。事实上，自 20 世纪 80 年代接受世界银行贷款开展卫生项目以来，中国从国际发展援助中获益良多。将中国作为一个发展中国家有效利用国际卫生发展援助的经验总结出来，即系统梳理中国接受对外援助过程时，中国和国际发展援助伙伴的具体做法，比如中国采取了哪些策略和做法以确保援助得以贯彻，国际发展援助伙伴又做出了哪些调整和让步使援助适用于中国的国情，并将其纳入受援国利用发展援助的最佳实践中去，将为中国今后支持其他发展中国家的卫生健康事业提供有益参考，对全球卫生也是一个重要贡献。

参考文献：

1. Jacobsen KH. Introduction to Global Health (2nd edition). Chapter 13: globalization and health. Burlington: Jones & Bartlett Learning; 2013.p.309-10.
2. Machalaba C, Romanelli C, Stoett P, Baum SE, Bouley TA, Daszak P, Karesh WB. Climate change and health: transcending silos to find solutions. Ann Glob Health. 2015; 81(3):445-8.
3. Benatar S, Poland B. Lessons for health from insights into environmental crises. Int J Health Serv. 2016;46(4):825-42.
4. The Millennium Development Goals Report 2015. https://www.undp.org/content/undp/en/home/librarypage/mdg/the-millennium-development-goals-report-2015.html. Accessed 27 Aug 2019.

5. Achieving Sustainable Health Development in the African Region Strategic Directions for WHO: 2010-2015. https://afro.who.int/sites/default/files/2018-03/strategic-directions2010-2015.pdf. Accessed 27 Aug 2019.
6. Noncommunicable Diseases Country Profiles 2018. https://www.who.int/nmh/publications/ncd-profiles-2018/en/.Accessed 27 Aug 2019.
7. Cherry J. The chronology of the 2002-2003 SARS mini pandemic. Paediatr Respir Rev. 2004;5(4):262-9.
8. Slemp C. Learning from the 2009 H1N1 pandemic and looking forward. West Virginia Medical Journal. 2010;106(5):34-5.
9. Bempong NE, Ruiz DC, Schütte S, Bolon I, Keiser O, Escher G, Flahault A. Precision global health - The case of Ebola: A scoping review. Journal of Global Health. 2019;DOI: 10.7189/jogh.09.010404.
10. Su S, Wong G, Liu Y, Gao GF, Li S, Bi Y. MERS in South Korea and China: a potential outbreak threat? Lancet. 2015;385(9985):2349-50.
11. Baud D, Gubler DJ, Schaub B, Lanteri MC, Musso D. An update on Zika virus infection. Lancet. 2017; DOI: 10.1016/S0140-6736(17)31450-2.
12. Interagency Coordination Group (IACG), No Time to Wait: Securing the future from drug-resistance infections, Report to the Secretary General of the United Nations. https://www.who.int/antimicrobial-resistance/interagency-coordination-group/final-report/en/. Accessed 27 Aug 2019.
13. Gale RP, Armitage JO. Are we prepared for nuclear terrorism? N Engl J Med. 2018;378(13):1246-54.
14. Hunter MD, Hunter JC, Yang JE, Crawley AW, Aragón TJ. Public health system response to extreme weather events. Journal of Public Health Management & Practice. 2016; 22(1):E1-10.
15. Moon S, Omole O. Development assistance for health: critiques, proposals and prospects for change. Health Economics Policy & Law. 2017;12(2):207-21.
16. Li H, Liu K, Gu J, Zhang Y, Qiao Y, Sun X. The development and impact of primary health care in China from 1949 to 2015: A focused review. Int J Health Plann Mgmt. 2017;32:339–50.
17. Guo Y, Bai J, Na H: The history of China's maternal and child health care development. Semin Fetal Neonatal Med. 2015;20(5):309-314.
18. 中华人民共和国国务院新闻办公室网站:《中国的医疗卫生事业》白皮书(全文). http://www.scio.gov.cn/ztk/dtzt/93/3/Document/1261899/1261899.htm
19. 中华人民共和国国务院新闻办公室网站:《中国的对外援助（2014）》白皮书(全文). http://www.scio.gov.cn/ztk/dtzt/34102/35574/35582/Document/1534198/1534198.htm.
20. Han Q, Chen L, Evans T, Horton R. China and global health. Lancet. 2008;372(9648):1439-41.
21. Ren M, Lu G. China's global health strategy. Lancet. 2014;384(9945):719-21.
22. Richards T. UK launches initiative on global health. BMJ. 2000;320(7232):402.
23. Martin MK: A UK global health strategy: the next steps. BMJ. 2007;335(7611):110.
24. Jacqui W: UK steps up its global health security. Lancet Infect Dis. 2008, 8(6):350.

25. Coltart CEM, Black ME, Easterbrook PJ. Global health in the UK government and university sector. Infect Dis Clin North Am. 2011;25(3):555-74.
26. Mwatsama MK, Wong S, Ettehad D, Watt NF. Global health impacts of policies: lessons from the UK. Globalization & Health. 2014;10(1):13.
27. Government HM: Health is global: a UK Government strategy 2008–2013. https://webarchive.nationalarchives.gov.uk/20130105191920/http://www.dh.gov.uk/en/Publicationsandstatistics/Publications/PublicationsPolicyAndGuidance/DH_088702. Accessed 27 Aug 2019.
28. Gagnon ML, Labonté R. Understanding how and why health is integrated into foreign policy - a case study of health is global , a UK Government Strategy 2008-2013. Globalization & Health. 2013;9(1):1-19.
29. Department for International Development - GOV.UK. https://www.gov.uk/government/organisations/department-for-international-development. Accessed 27 Aug 2019.
30. Emerging powers--Speech by Andrew Mitchell at the Royal Institute of International Affairs. 2011. https://www.gov.uk/government/speeches/emerging-powers. Accessed 27 Aug 2019.
31. 中共中央国务院印发《"健康中国2030"规划纲要》. http://www.gov.cn/gongbao/content/2016/content_5133024.htm. Accessed 27 Aug 2019.
32. 国务院关于印发"十三五"卫生与健康规划的通知. http://www.gov.cn/zhengce/content/2017-01/10/content_5158488.htm. Accessed 27 Aug 2019.
33. Liu P, Guo Y, Qian X, Tang S, Li Z, Chen L: China's distinctive engagement in global health. Lancet. 2014;384(9945):793-804.
34. Shajalal M, Xu J, Jing J, King M, Zhang J, Wang P, Bouey J, Cheng F. China's engagement with development assistance for health in Africa. Glob Health Res Policy. 2017;DOI: 10.1186/s41256-017-0045-8.
35. 中华人民共和国外交部. 变革我们的世界：2030年可持续发展议程. https://www.fmprc.gov.cn/web/ziliao_674904/zt_674979/dnzt_674981/qtzt/2030kcxfzyc_686343/t1331382.shtml. Accessed 27 Aug 2019.
36. Triangular Co-operation: What's the literature telling us? (2013). http://www.oecd.org/dac/dac-global-relations/oecdpublicationsontriangularco-operation.htm. Accessed 27 Aug 2019.
37. 中华人民共和国国家主席习近平：携手共命运 同心促发展——在二〇一八年中非合作论坛北京峰会开幕式上的主旨讲话 http://www.gov.cn/gongbao/content/2018/content_5323084.htm. Accessed 27 Aug 2019.
38. "一带一路"国际合作高峰论坛圆桌峰会联合公报. http://www.gov.cn/xinwen/2019-04/27/content_5386929.htm. Accessed 27 Aug 2019.
39. 健康中国行动(2019—2030年）. http://www.gov.cn/xinwen/2019-07/15/content_5409694.htm. Accessed 27 Aug 2019.
