# Supplementary material for: China-UK partnership for global health: practices and implications of the Global Health Support Programme 2012–2019
Source: Glob Health Res Policy. 2020 Mar 20;5:13. doi: 10.1186/s41256-020-00134-7 (PMC7083009; doi:10.1186/s41256-020-00134-7)
Supplement: Supplementary file 2 — Additional file 2. The Main Academic Outputs and Deliverables Produced by the GHSP. [file 41256_2020_134_MOESM2_ESM.pdf]

Additional file 2 The Main Academic Outputs and Deliverables Produced by the GHSP

Additional file 2-1 Research Reports

No.	Name of Research Report	Code of Outputs
**1. Series of Reports of China Global Health Strategy**		
1.	1.1 Fundamental theoretical and practical research report of China global health strategy	OP3
2.	1.2 Sub-research of China global health strategy-Comparative research of OECD national global health strategy	OP3
3.	1.3 Sub-research of China global health strategy-infectious diseases and neglected tropical diseases	OP3
4.	1.4 Sub-research of China global health strategy-trade and health	OP3
5.	1.5 Sub-research of China global health strategy-health system	OP3
6.	1.6 Sub-research of China global health strategy-environment and health	OP3
7.	1.7 Sub-research of China global health strategy-promote health in whole life	OP3
8.	1.8 Sub-research of China global health strategy-health an food	OP3
9.	1.9 Sub-research of China global health strategy-diplomacy and health	OP3
10.	1.10 Sub-research of China global health strategy-chronic disease	OP3
11.	1.11 Sub-research of China global health strategy-health emergency	OP3
12.	1.12 Research completion report of China global health strategy	OP3
13.	1.13 Proposed China global health strategy	OP3
**2. Global Health Green Paper-China's experiences and strategies under the global health governance view (published)**[1]		
14.	2.1 China's Engagement in Global Health- based on "National Capacity" analysis perspective	OP3
15.	2.2 International Chapter: Strategy, Practice and Enlightenment of Global Health in BRICS Countries	OP3
16.	2.3 International Chapter: Civil society organizations and global health: international experiences and reference	OP3
17.	2.4 International Chapter: Trend, prevention development and health strategy research in China and 5 other Asian countries	OP3
18.	2.5 International Chapter: Decentralizing The Provision of Antiretroviral Therapy to People Living	OP3

[1] The reports listed in this category are 16 chapters in the published Global Health Green Paper.

No.	Name of Research Report	Code of Outputs
	with Hiv/Aids in Vietnam	
19.	2.6 International Chapter: China's Engagement in Global Health: Research on International Perceptions of Global Health and Chinese Medical Team Program	OP3
20.	2.7 National Chapter: Chronic disease prevention and control strategy and China action direction after 2015	OP3
21.	2.8 National Chapter: The experiences of China AIDS prevention and control and the roles and development strategies of China in global AIDS prevention and control in later Millennium Era	OP3
22.	2.9 National Chapter: The strategies of China TB transmission and control and its inspiration to developing countries-TB control strategies and methods expectation after 2015	OP3
23.	2.10 National Chapter: The inspiration and reference of China's experiences of schistosomiasis prevention and control to Africa	OP3
24.	2.11 National Chapter: China's Key Tasks of Participating in Global Health on Tropical Diseases in Post-2015	OP3
25.	2.12 National Chapter: The action direction and global health strategy of safe abortion in China after 2015	OP3
26.	2.13 National Chapter: Research report of children malnutrition intervention methods and implementation experiences in China	OP3
27.	2.14 National Chapter: Strategy research of essential medicine system progress and development in China	OP3
28.	2.15 National Chapter: Research report of smoking control in China-difficulties, shortages, and futural direction	OP3
29.	2.16 National Chapter: Response strategy to climate change health risks in China	OP3
**3. Other Research Reports on Global Health Governance**		
30.	3.1 Research report of category II and category III diseases relevant medicine product R&D status	OP3
31.	3.2 Global Governance on Antibiotics Resistance and the Revelations for China (Antibiotics Resistance)	OP3
32.	3.3 Global Governance and Key Countries' Participation in Antibiotics Resistance and the Revelations for China (Antibiotics Resistance)	OP3
33.	3.4 Research on Artemisinin Resistance in Yuci Region (Antibiotics Resistance)	OP3
**4. Development Assistance for Health– Researches on Development Assistance for Health provided by Traditional Donor Countries and International Organizations**		

No.	Name of Research Report	Code of Outputs
34.	4.1 Research report of international health development assistance-major methods, management system and emerging aid entities	OP2
35.	4.2 Training material for health development assistance	OP2
**5. Development Assistance for Health: Researches on Development Assistance for Health to China by Traditional Donor Countries and International Organizations**		
36.	5.1 Pragmatic and innovative, solve the highlighted health problem in receipt countries in exploration and practice- the road of China foreign aid from example of China-Gates TB programme	OP2
**6. Development Assistance for Health: Researches on China's Foreign Aid and South-South Cooperation**		
37.	6.1 Summary and assessment research of Chinese foreign health assistance	OP2
38.	6.2 The anti-malaria assessment research report of China aid to Africa	OP2
39.	6.3 The effectiveness assessment of health development aid from China to Uganda	OP2
40.	6.4 Bilateral and fourfold embedment, the development aid for health from China to Uganda (literature review)	OP2
41.	6.5 Host and guest: Jinja hospital and China medical team (Jinja hospital ethnography)	OP2
42.	6.6 Case analysis of foreign aid medical development of Jiangsu	OP2
43.	6.7 Evaluation of Chinese medical team in Tanzania	OP2
44.	6.8 Evaluation of the Fast Elimination of Malaria by Source Eradication (FEMSE) in Comoros	OP2
45.	6.9 Cooperation model on schistosomiasis prevention and control between China and other Asian countries	OP2
46.	6.10 Major experiences and lessons to carry out malaria project in Myanmar by Health Fight Against Poverty Action Yunnan Office	OP2
47.	6.11 tatus of primary care and MCH assistance project in Parkistan and areas and patterns for China - Parkistan health development collaboration	OP2
48.	6.12 A Review of the Best practices of South-South Health Cooperation and their policy Implications	OP2
**7. Development Assistance for Health: Researches on the Belt and Road Initiative**		
49.	7.1 Health Collaboration Needs Assessment and the Collaborating Strategy Research of Vietnam and Laos	OP2
50.	7.2 Health Collaboration Needs Assessment and the Collaborating Strategy Research of Cambodia and Myanmar along the Belt and Road	OP2
51.	7.3 Health Collaboration Needs Assessment and the Collaborating Strategy Research on Neglected	OP2

No.	Name of Research Report	Code of Outputs
	Zoonotic Diseases in Key Countries along the Belt and Road: A Case Study of Echinococcosis Prevention and Control in Mongolia	
52.	7.4 Research on Traditional Chinese Medicine Trade and Exchange Cooperation Promotion in China-Thailand and China-Philippines	OP2
53.	7.5 Research on the Needs Assessment and Cooperative Strategies for Infectious Disease Prevention and Control in China-Malaysia and China-Cambodia	OP2
54.	7.6 Research on the Needs Assessment and Cooperative Strategies for Health Cooperation in China-Mongolia and China-Russia	OP2
55.	7.7 Research on the Cooperation Strategy of Transnational Communication of TCM Culture: A Case Study of Nepal	OP2
56.	7.8 Research on the Cooperation Strategy of China-ASEAN Health Human Resources: A Case Study of the Primary Health Care Capacity Construction of ASEAN 10+3 Nursing Coalition	OP2
**8. China's Experience: Health System Strengthening**		
57.	8.1 Sub-report of Research on Health Systems Strengthening in China: Overview	OP1
58.	8.2 Sub-report of Research on Health Systems Strengthening in China: Its Evolution and Expression in Specific Health Policies	OP1
59.	8.3 Sub-report of Research on Health Systems Strengthening in China: Financing Strategies for Universal Health Coverage: Evidence from Rural Health Insurance in China	OP1
60.	8.4 Sub-report of Research on Health Systems Strengthening in China: Financing Strategies to Improve Access to Public Health	OP1
61.	8.5 Sub-report of Research on Health Systems Strengthening in China: Evolution, Structure, and Function of the Three-tiered Health Service Delivery System in Rural China	OP1
62.	8.6 Sub-report of Research on Health Systems Strengthening in China: Development and Evolution of Community Health Workers in Rural China	OP1
**9. China's Experience: Maternal and Child Health**		
63.	9.1 Research Report on policy-making process and historical summary of China's Maternal and Child Health	OP1
64.	9.2 Research Report on maternal and child safety	OP1
65.	9.3 Research Report on diseases interception from maternal to child	OP1
66.	9.4 Research Report on Children Nutrition	OP1
**10. China's Experience: Disease Prevention and Control**		

No.	Name of Research Report	Code of Outputs
67.	10.1 Status of schistosomiasis control products and demands report: evaluation report of national schistosomiasis immunodiagnostic reagents	OP1
68.	10.2 A systematic review of oncomelania drugs	OP1
**11. China's Experience: Researches on Country Context Analysis and Feasibility**		
69.	11.1 Report of Context Comparative Analysis on China, Vietnam, Lao People's Democratic Republic and Ghana	OP1
70.	11.2 China Context Analysis Report	OP1
71.	11.3 Vietnam Context Analysis Report	OP1
72.	11.4 Lao People's Democratic Republic Context Analysis Report	OP1
73.	11.5 Ghana Context Analysis Report	OP1
74.	11.6 Transferring health system policies and interventions across settings – a literature review and framework	OP1
75.	11.7 Addressing challenges in human resource and health financing for the poor and informal sector in Tanzania with experience from China: A comparative study	OP1
76.	11.8 Feasibility Study on Applying China's Experience in Malaria Prevention and Control in Tanzanian	OP1
77.	11.9 Enhancing collaboration between the People's Republic of China and African countries for schistosomiasis control	OP1
78.	11.10 Approaches in scaling up schistosomiasis intervention towards elimination in Africa: leveraging from the Chinese experience and lessons	OP1
79.	11.11 Opportunities and Challenges for China to Participating in controlling schistosomomiasis & malaria in Africa	OP1
**12. China's Experience: Implementing Proposal**		
80.	12.1 HIV prevention from mother to child in Vietnam: Barriers to access and utilization of services and measures	OP 1
81.	12.2 Intervention model for enhancing and maintaining the sustainability of the implementation of 10 steps to successful breastfeeding in some hospitals in Vietnam	OP 1
82.	12.3 Role of village health workers network to promote institutional based childbirth in rural mountainous areas in Vietnam	OP 1
83.	12.4 Supportive Supervision to Strengthen Maternal and Child Health in Ghana	OP 1
84.	12.5 Improving child nutrition in rural communities in saravan province, Lao PDR: Adopting China	OP1

No.	Name of Research Report	Code of Outputs
	experience	
85.	12.6 Health workers' capacity in provision of skilled childbirth care at Vientiane Capital health centers: Adopting China experience	OP1
86.	12.7 Pilot project proposal-OP402 pilot project on Malaria in Tanzania	OP1
**13. Others**		
87.	Translation collection of the global health strategies from the main countries of the world	OP3

Additional file 2-2 Books (including chapters)

No.	Name of Book[2]	Main Author	Employer of Author	Name of Press	Publish Date	Remarks	Code of Outputs
1.	Innovative health partnership – the diplomacy of diversity (translation)	GUO Yan	School of Public Health, Peking University	Peking University Medical Press	April 2014	Compiled in 《Global Health Diplomacy》	OP3
2.	Negotiating and navigating global health – case studies in global health diplomacy (translation)	GUO Yan	School of Public Health, Peking University	Peking University Medical Press	August 2014	Compiled in 《Global Health Diplomacy》	OP3
3.	21st century Global health diplomacy (translation)	GUO Yan	School of Public Health, Peking University	Peking University Medical Press	March 2017	Compiled in 《Global Health Diplomacy》	OP3
4.	Introduction to global health	GUO Yan, LIU Peilong	School of Public Health, Peking University	Peking University Medical Press	Forthcomin g	Compiled in 《Peking University Health Science Center Preventive Medicine Textbook Series》	OP2
5.	Case Study on Promotion of Maternal and Child Health and Child Nutrition in China	QIAN Xu	Global Health Institute, Fudan University	Fudan University Press	April 2017		OP1
6.	Global health green paper-China's	YANG Xiaoguang	School of Public Health, Fudan	Fudan University Press	November 2017		OP1

[2] Considering the book publishing cycle, this list includes books to be published.

No.	Name of Book[2]	Main Author	Employer of Author	Name of Press	Publish Date	Remarks	Code of Outputs
	experiences and strategies under the global health governance view	CHEN Wen	University				
7.	China-Africa collaboration-international development assistance theory exploration and innovation - Chapter Universal health coverage funding: experiences, lessons and inspiration from China	WANG Yunping	China National Health Development Research Center, National Health Commission	People’s Medical Publishing House	November 2015	Compiled in 《China South-South Health Cooperation Research Series》	OP2
8.	Environment and Global Health	KAN Haidong , LU Yuanan	School of Public Health, Fudan University	People's Medical Publishing House	2016	Partially supported by GHSP	OP3
9.	Surveillance and Management of Parasitic Diseases	ZHOU Xiaonong	National Institute of Parasitic Diseases，China CDC	People's Medical Publishing House	September 2017	Compiled in 《Human Parasitic Disease Prevention and Control Series》	OP1
10.	Make friends first: a case study of development aid for health from China to Uganda	QIU Zeqi	Center for China Social and Development Research, Peking University	Social Sciences Academic Press	February 2017		OP2
11.	Annual report on China’s international security studies (2016)	WANG Yunping	China National Health Development	Social Science Academic Press	December 2017	Compiled in 《Blue Book of International	OP3

No.	Name of Book[2]	Main Author	Employer of Author	Name of Press	Publish Date	Remarks	Code of Outputs
	- Chapter Chinese public health safety and major power responsibility under global view		Research Center, National Health Commission			Security》	
12.	African Religion Development Report (2015-2016) - Chapter African public health governance development annual report (2015-2016)	WANG Yunping	China National Health Development Research Center, National Health Commission	China Social Sciences Press	January 2017	Compiled in 《Philosophy and Social Science Development Report of Ministry of Education》	OP2
13.	Strategy study on health collaboration based on one belt and one road initiative: case study from Vietnam and Laos	HUANG Jiayan	School of Public Health, Fudan University	Fudan University Press	2018		OP2
14.	Evaluation of the Fast Elimination of Malaria by Source Eradication in the Union of Comoros	Zheng Xie	School of Public Health, Peking University	Springer	Forthcomin g	Published in English	OP2

Additional file 2-3 Journal Papers

No[3].	Title	First Author and His/Her Affiliation	Journal Information[4]	Code of Outputs
1.	Progress in research, control and elimination of helminth infections in Asia	Jürg Utzinger, etc. Department of Epidemiology and Public Health, Swiss Tropical an Public Health Institute	**ActaTropica**，2015,141:135-145	**OP1**
2.	Historical Patterns of Malaria Transmission in China.	Jian-Hai Yin, etc.National Institute of Parasitic Diseases，China CDC	**Advances in Parasitology -** Malaria Control and Elimination Programme in China，2014: 1-15	**OP1**
3.	Feasibility and Roadmap Analysis for Malaria Elimination in China.	Xiao-Nong Zhou, etc.National Institute of Parasitic Diseases，China CDC	**Advances in Parasitology -** Malaria Control and Elimination Programme in China，2014: 21-42	**OP1**
4.	Lessons from Malaria Control to Elimination: Case Study in Hainan and Yunnan Provinces.	Zhi-Gui Xia, etc.National Institute of Parasitic Diseases，China CDC	**Advances in Parasitology -** Malaria Control and Elimination Programme in China，2014: 47-76	**OP1**
5.	Surveillance and Response to Drive the National Malaria Elimination Program.	Xin-Yu Feng, etc.National Institute of Parasitic Diseases，China CDC	**Advances in Parasitology -** Malaria Control and Elimination Programme in China，2014: 81-104	**OP1**
6.	Operational Research Needs Toward Malaria Elimination in China.	Shen-Bo Chen, etc.National Institute of Parasitic Diseases，China CDC	**Advances in Parasitology -** Malaria Control and Elimination Programme in China: 109-127	**OP1**
7.	Approaches to the Evaluation of Malaria Elimination at County Level: Case Study in the Yangtze River Delta Region.	Min Zhu, etc.Shanghai Municipal CDC	**Advances in Parasitology -** Malaria Control and Elimination Programme in China，2014: 135-180	**OP1**
8.	Surveillance and Response Strategy in the Malaria Post-elimination Stage: Case Study of Fujian Province.	Fa-Zhu Yang, etc.Fujian Province CDC	**Advances in Parasitology -** Malaria Control and Elimination Programme in China，2014: 183-202	**OP1**

[3] Paper number from 1 to 57 are published in international journals, while papers number from 58 to 126 are published in domestic journals.
[4] The published papers are sorted in ascending order according to the names of the journals.

No[3].	Title	First Author and His/Her Affiliation	Journal Information[4]	Code of Outputs
9.	Preparation of Malaria Resurgence in China: Case Study of Vivax Malaria Re-emergence and Outbreak in Huang-Huai Plain in 2006.	Hong-Wei Zhang, etc.Henan Province CDC	**Advances in Parasitology -** Malaria Control and Elimination Programme in China，2014: 205-226	**OP1**
10.	Preparedness for Malaria Resurgence in China: Case Study on Imported Cases in 2000–2012.	Jun Feng, etc.National Institute of Parasitic Diseases，China CDC	**Advances in Parasitology -** Malaria Control and Elimination Programme in China，2014: 231-261	**OP1**
11.	Preparation for Malaria Resurgence in China: Approach in Risk Assessment and Rapid Response.	Ying-Jun Qian, etc. National Institute of Parasitic Diseases，China CDC	**Advances in Parasitology -** Malaria Control and Elimination Programme in China，2014: 267-283	**OP1**
12.	Transition from Control to Elimination: Impact of the 10-Year Global Fund Project on Malaria Control and Elimination in China	Ru-Bo Wang, etc. National Institute of Parasitic Diseases，China CDC	**Advances in Parasitology -** Malaria Control and Elimination Programme in China，2014: 289-317	**OP1**
13.	China–Africa Cooperation Initiatives in Malaria Control and Elimination.	Zhi-Gui Xia, etc. National Institute of Parasitic Diseases，China CDC	**Advances in Parasitology -** Malaria Control and Elimination Programme in China，2014: 319-335	**OP2**
14.	Evolution of the National Schistosomiasis Control Programmes in The People's Republic of China	J. Xu, etc. National Institute of Parasitic Diseases，China CDC	**Advances in Parasitology -** Schistosomiasis in The People's Republic of China: from control to Elimination，2016: 1-29	**OP1**
15.	Epidemiological Features and Effectiveness of Schistosomiasis Control Programme in Lake and Marshland Region in The People's Republic of China	S.-Q. Zhang, etc.Anhui Provincial Institute of Schistosomiasis Control	**Advances in Parasitology -** Schistosomiasis in The People's Republic of China: from control to Elimination，2016: 39-67	**OP1**
16.	Epidemiological Features and Effectiveness of Schistosomiasis Control Programme in Mountainous and Hilly Region of The People's Republic of China	Y. Liu, etc. Sichuan Province CDC	**Advances in Parasitology -** Schistosomiasis in The People's Republic of China: from control to Elimination，2016: 73-91	**OP1**
17.	Epidemiological Features and Control Progress of Schistosomiasis in Waterway- Network Region in The People's Republic of China	L. Shi, etc. Jiangsu Institute of Parasitic Diseases	**Advances in Parasitology -** Schistosomiasis in The People's Republic of China: from control to	**OP1**

No[3].	Title	First Author and His/Her Affiliation	Journal Information[4]	Code of Outputs
			Elimination，2016: 97-114	
18.	The Establishment and Function of Schistosomiasis Surveillance System Towards Elimination in The People's Republic of China	L.-J. Zhang, etc. Key Laboratory of Parasite and Vector Biology of the Chinese Ministry of Health	**Advances in Parasitology -** Schistosomiasis in The People's Republic of China: from control to Elimination，2016: 117-137	**OP1**
19.	Applications of Spatial Technology in Schistosomiasis Control Programme in The People's Republic of China	X.-Y. Wang, etc. Jiangsu Institute of Parasitic Diseases	**Advances in Parasitology -** Schistosomiasis in The People's Republic of China: from control to Elimination，2016: 143-159	**OP1**
20.	Reaching the Surveillance- Response Stage of Schistosomiasis Control in The People's Republic of China: A Modelling Approach	Y. Feng, etc. Key Laboratory of National Health and Family Planning Commission on Parasitic Disease Control and Prevention	**Advances in Parasitology -** Schistosomiasis in The People's Republic of China: from control to Elimination，2016: 165-188	**OP1**
21.	Biology and Control of Snail Intermediate Host of Schistosoma japonicum in The People's Republic of China	Z.-J. Li, etc. Jiangxi Provincial Institute of Schistosomiasis Control	**Advances in Parasitology -** Schistosomiasis in The People's Republic of China: from control to Elimination，2016: 197-228	**OP1**
22.	Integrated Control Strategy of Schistosomiasis in The People's Republic of China: Projects Involving Agriculture, Water Conservancy, Forestry, Sanitation and Environmental Modification	Y. Yang, etc. School of Public Health, Fudan University	**Advances in Parasitology -** Schistosomiasis in The People's Republic of China: from control to Elimination，2016: 237-262	**OP1**
23.	Towards the Elimination of Schistosomiasis japonica through Control of the Disease in Domestic Animals in The People's Republic of China: A Tale of over 60 Years	Z.-G. Cao, etc. Department of Pathogenic Biology and Immunology, Xi'an Jiaotong University Health Science Center	**Advances in Parasitology -** Schistosomiasis in The People's Republic of China: from control to Elimination，2016: 269-302	**OP1**
24.	Health Education as an Important Component in the National Schistosomiasis Control Programme in The People's Republic of China	L. Chen, etc. Sichuan Province CDC	**Advances in Parasitology -** Schistosomiasis in The People's Republic of China: from control to Elimination，2016: 307-333	**OP1**
25.	Policy Support and Resources Mobilization for the	H. Zhu, etc. Hebei Province CDC	**Advances in Parasitology -**	**OP1**

No[3].	Title	First Author and His/Her Affiliation	Journal Information[4]	Code of Outputs
	National Schistosomiasis Control Programme in The People's Republic of China		Schistosomiasis in The People's Republic of China: from control to Elimination，2016: 341-378	
26.	New Anti-Schistosoma Approaches in The People's Republic of China: Development of Diagnostics, Vaccines and Other New Techniques Belonging to the 'Omics' Group	S.-B. Chen, etc. National Institute of Parasitic Diseases，China CDC	**Advances in Parasitology -** Schistosomiasis in The People's Republic of China: from control to Elimination，2016: 385-404	**OP1**
27.	Development and Application of Diagnostics in the National Schistosomiasis Control Programme in The People's Republic of China	J.-F. Zhang, etc. Zhejiang Academy of Medical Sciences	**Advances in Parasitology -** Schistosomiasis in The People's Republic of China: from control to Elimination，2016: 409-428	**OP1**
28.	China-Africa and China-Asia Collaboration on Schistosomiasis Control: A SWOT Analysis	J. Xu, etc. National Institute of Parasitic Diseases，China CDC	**Advances in Parasitology -** Schistosomiasis in The People's Republic of China: from control to Elimination，2016: 435-457	**OP2**
29.	Strengthening maternal and child health in China: Lessons from transforming policy proposals into action	Xiaoguang Yang, etc. School of Public Health, Fudan University	**BioScience Trends**，2018,12(2):212-214	**OP1**
30.	Towards universal access to skilled birth attendance: the process of transforming the role of traditional birth attendants in Rural China	Hong Jiang, etc. Global Health Institute, Fudan University	**BMC Pregnancy and Childbirth**，2016,16:58-66	**OP1**
31.	Lifestyle factors associated with childhood obesity: a cross-sectional study in Shanghai, China	Liangli Li, etc. School of Public Health, Fudan University	**BMC Research Notes**，2015, 8(1): 6-13	**OP1**
32.	Associations between size-fractionated particulate air pollution and blood pressure in a panel of type II diabetes mellitus patients	Ang Zhao, etc. School of Public Health, Fudan University	**Environment International**，2015, 80: 19-25	**OP1**
33.	Ambient air pollution, temperature and out-of-hospital coronary deaths in Shanghai, China	Jinping Dai, etc. Shandong Provincial Environmental Monitoring Center Station	**Environmental Pollution**，2015, 203: 116-121	**OP1**
34.	Genetic diversity of the Mycobacterium tuberculosis Beijing family based on multiple genotyping profiles	Y. LIU, etc. School of Public Health, Fudan University	**Epidemiology and Infection**，2016, 144(08): 1728-1735	**OP1**

No[3].	Title	First Author and His/Her Affiliation	Journal Information[4]	Code of Outputs
35.	Size-fractionated Particulate Air Pollution and Circulating Biomarkers of Inflammation, Coagulation, and Vasoconstriction in a Panel of Young Adults	Renjie Chen, etc. School of Public Health, Fudan University	**Epidemiology**，2015, 26(3): 328-336.	**OP1**
36.	The production and sales of anti-tuberculosis drugs in China	Yang-Mu Huang, etc. School of Public Health, Peking University	**Infectious Diseases of Poverty**, 2016,5: 88	**OP1**
37.	Bibliometric study of research and development for neglected diseases in the BRICS	Jing Bai. etc. School of Public Health, Peking University	**Infectious Diseases of Poverty**, 2016,5: 89	**OP1**
38.	Effectiveness and impact of the crossborder healthcare model as implemented by non-governmental organizations: case study of the malaria control programs by health poverty action on the China-Myanmar border	Jun Zhang, etc. Health Poverty Action	**Infectious Diseases of Poverty**, 2016,5:80	**OP2**
39.	Lessons on malaria control in the ethnic minority regions in Northern Myanmar along the China border, 2007–2014	Ru-Bo Wang, etc. National Institute of Parasitic Diseases，China CDC	**Infectious Diseases of Poverty**, 2016,5:95	**OP2**
40.	Domestic trends in malaria research and development in China and its global influence	Yang-Mu Huang, etc. School of Public Health, Peking University	**Infectious Diseases of Poverty**, 2017,6:4	**OP1**
41.	Application of Community-Based and Integrated Strategy to Reduce Malaria Disease Burden in Southern Tanzania: the study protocol of China-UK-Tanzania Pilot Project of Malaria Control	Duo-Quan Wang, etc. National Institute of Parasitic Diseases，China CDC	**Infectious Diseases of Poverty, 2019, 8(1):4**	**OP4**
42.	The role of health system governance in strengthening the rural health insurance system in China	Beibei Yuan, etc. School of Public Health, Peking University	**International Journal for Equity in Health**，2017,16(44):1-20	**OP1**
43.	Extending access to essential services against constraints: the three-tier health service delivery	Xing Lin Feng, etc. School of Public Health, Peking University	**International Journal for Equity in Health**，2017,16(44):21-38	**OP1**

No[3].	Title	First Author and His/Her Affiliation	Journal Information[4]	Code of Outputs
	system in rural China (1949–1980)			
44.	Development of village doctors in China: financial compensation and health system support	Dan Hu, etc. China Center for Health Development Studies, Peking University	**International Journal for Equity in Health**, 2017,16(44):39-45	**OP1**
45.	Impact of health workforce availability on health care seeking behavior of patients with diabetes mellitus in China	Yinzi Jin, etc. China Center for Health Development Studies, Peking University	**International Journal for Equity in Health**, 2017,16(44):46-55	**OP1**
46.	Financing strategies to improve essential public health equalization and its effects in China	Li Yang, etc. China Center for Health Development Studies, Peking University	**International Journal for Equity in Health**, 2017,16(44):56-67	**OP1**
47.	Strengthening health system to improve immunization for migrants in China	Hai Fang, etc. China Center for Health Development Studies, Peking University	**International Journal for Equity in Health**, 2017,16(44):68-77	**OP1**
48.	Challenges for gatekeeping: a qualitative systems analysis of a pilot in rural China	Jin Xu, etc. China Center for Health Development Studies, Peking University	**International Journal for Equity in Health**, 2017,16(44):78-98	**OP1**
49.	The acute effects of outdoor temperature on blood pressure in a panel of elderly hypertensive patients	Renjie Chen, etc. School of Public Health, Fudan University	**International Journal of Biometeorology**, 2015, 59(12): 1791-1797	**OP1**
50.	Barriers and enablers of the prevention of mother to child transmission of HIVAIDS program in China	Zhuoxin Peng, etc. Department of Epidemiology, Fudan University	**International Journal of Infectious Diseases**, 2017:72–80	**OP1**
51.	China-Africa Health Development Initiatives: Benefits and Implications for Shaping Innovative and Evidence-informed National Health Policies and Programs in Sub-saharan African Countries	Ernest Tambo, etc. Africa Disease Intelligence and Surveillance, Communication and Response Foundation	**International Journal of MCH and AIDS**, 2016,5(2):119-133	**OP2**
52.	The production and exportation of artemisinin-derived drugs in China: current status and existing challenges	Yangmu Huang, etc. School of Public Health, Peking University	**Malaria Journal**, 2016,15:365	**OP1**
53.	Epidemiological characterization of malaria in rural southern Tanzania following China-Tanzania pilot joint malaria control baseline survey	Rashid A. Khatib, etc. Ifakara Health Institute, Tanzania	**Malaria Journal**, 2018,17:292-305	**OP4**
54.	Increasing Coverage of Hepatitis B Vaccination in China	Shengnan Wang, etc. School of Public Health, Fudan University	**Medicine**, 2016,95(19):1-15	**OP1**
55.	A pilot study on the simulation-based training for	Ying Zhao, etc. School of Nursing, Fudan	**Nurse Education in Practice**, 2019	**OP4**

No[3].	Title	First Author and His/Her Affiliation	Journal Information[4]	Code of Outputs
	Ethiopia skilled birth attendants	University	(34):130-138	
56.	Socioeconomic Status and Physical Activity in Chinese Adults: A Report from a Community-Based Survey in Jiaxing, China	Mingling Chen, etc. School of Public Health, Fudan University	**PLOS ONE**, 2015, 10(7): e132918	**OP1**
57.	Challenges of research and development on antimalarial medicinal products in China: a bibliometric analysis and systematic review	Rui She, etc. School of Public Health, Peking University	**Transactions of the Royal Society of Tropical Medicine and Hygiene**, 2016, 110 (11): 649	**OP1**
58.	Social determinants of growth retardation in children under 5 years of age in China	Mansailimai, etc. School of Public Health, Peking University	Journal of Peking University (Health Sciences) , 2016,48(3):418-423	OP1
59.	Decode the China symbols from health assistance to Africa	Zeqi Qiu, etc. Center for Social Research, Peking University	Africa Research, 2017,01:49-72	OP2
60.	Development process of chronic disease prevention and control in China	Xiaona Liu, etc. School of Public Health, Fudan University	Journal of Public Health and Preventive Medicine, 2015,26(02):79-83	OP1
61.	Evolution of China foreign health aid decision making and management mechanism	Peilong Liu, etc. School of Public Health, Peking University	The Journal of International Studies, 2015,(2): 61-72	OP2
62.	Diplomacy transition and global health diplomacy	Qingmin Zhang. School of International Studies, Peking University	The Journal of International Studies, 2015,2:11-34.	OP3
63.	Research Advance on Health Effects of Secondhand Smoke Exposure in Occupational Groups	Xinyuan Huang, etc. School of Public Health, Fudan University	Journal of Environmental and Occupational Medicine, 2016,33(04):403-407	OP3
64.	Review on Interventions and Practice of Tobacco Control in China, 2006-2015	Xinyuan Huang, etc. School of Public Health, Fudan University	Journal of Environmental and Occupational Medicine, 2016,33(05):461-465	OP1
65.	How to integrate health collaboration into “One Belt, One Road”	Hongwei Yang. China National Health Development Research Center, National Health Commission	Health News	OP2
66.	Health collaboration of “One Belt, One Road” should abide by 5 principles	Yunping Wang. China National Health Development Research Center, National Health Commission	Health News	OP2
67.	Combat against Ebola: China won the world respect	Yunping Wang. China National Health Development Research Center, National Health	Health News	OP2

No[3].	Title	First Author and His/Her Affiliation	Journal Information[4]	Code of Outputs
		Commission		
68.	The “Expectation” in “Suspension”: A study about the Daily Life of Chinese Medical Team in Tanzania	Muye Tian, etc. China Agricultural University	Qinghai Journal of Ethnology , 2017,28(2):11-17	OP2
69.	View the global public health safety issue from Ebola epidemic	Yunping Wang. China National Health Development Research Center, National Health Commission	Current information online	OP3
70.	Malaria prevent knowledge and its influencing factors among travelers to Africa	Yuan Yuan, etc. School of Public Health, Peking University	Modern Preventive Medicine，2015, 42(7):1254-1256	OP3
71.	Intervention methods and implementation experiences of Chinese children malnutrition	Junfei Guo, etc.School of Public Health, Fudan University	Chinese Journal of Child Health Care，2015,23(07):724-726	OP1
72.	Literature review on implementation and effectiveness of health care services after abortion in China	Wei Yang, etc.School of Public Health, Fudan University	Maternal and Child Health Care of China，2015,30(32):5717-5721	OP1
73.	Coordination of development assistance for health at global and country levels	Kun Tang, etc.<br>School of Public Health, Peking University	Chinese Journal of Public Health Management，2016, 32(5): 632-638	OP2
74.	Features of two large －scale international health assistance programs for China	Honegmei Li, etc. National Institute of Parasitic Diseases，China CDC	Chinese Journal of Public Health Management，2018,34(2):148-151	OP2
75.	Gaps in clonorchiasis control in China	Menbao Qian, etc. National Institute of Parasitic Diseases，China CDC	Chinese Journal of Parastiology and Parasitic Diseases，2016, 34(4):373-376	OP1
76.	An overview on cooperation strength between China and the Global Fund to Fight AIDS, Tuberculosis and Malaria in developing multilateral malaria control projects	Zhigui Xia, etc.National Institute of Parasitic Diseases，China CDC	Chinese Journal of Parastiology and Parasitic Diseases，2017,35(6):520-526.	OP2
77.	Summary of domestic and international smoking control legislation implementation in public places	Zhiyan Zhu, etc. School of Public Health, Fudan University	Chinese Journal of Health Education，2015,31(10):964-968	OP3
78.	Research progress on broad smoking rationalizations	Xinyuan Huang, etc. School of Public Health, Fudan University	Chinese Journal of Health Education，2015,31(12):1168-1172	OP3
79.	Health economics assessment on frequently-used strategies and methods for chronic diseases	Xiaoyu Liu, etc. School of Public Health, Fudan University	Chinese Journal of Prevention and Control of Chronic Diseases，2015,23(11):865-869	OP3
80.	Inspiration of typical cases in prevention and control	Xiaona Liu, etc. School of Public Health, Fudan	Chinese Journal of Prevention and	OP1

No[3].	Title	First Author and His/Her Affiliation	Journal Information[4]	Code of Outputs
	of chronic non-communicable disease in China	University	Control of Chronic Diseases, 2016,24(03):169-174	
81.	Comparation and policy inspiration of chronic disease prevention and control strategies between China and Japan	Wanghong Xu, etc.<br>School of Public Health, Fudan University	Chinese Journal of Prevention and Control of Chronic Diseases, 2016,24(08):593-596	OP1
82.	A retrospective analysis on the effectiveness of falciparum malaria joint control in Jiangsu, Anhui and Henan provinces	Xiangli Kong, etc. Shandong Institute of Parasite Diseases	China Tropical Medicine , 2016,16(10):986-988	OP1
83.	Comparative Research on BRICS' Investment in Research and Development on Neglected Diseases	Jing Bai, etc. School of Public Health, Peking University	Chinese Health Economics, 2016,35(10):61-64.	OP3
84.	The accounting and analysis on the research and development of government expenditure on health in China	Yunping Wang, etc. China National Health Development Research Center, National Health Commission	Chinese Health Economics, 2016,35(2):38-41	OP1
85.	Introduction and inspiration of recruitment system for doctors without borders foreign aid medical team	Jingmiao Long, etc. School of Public Health, Peking University	China Health Human Resources, 2017, 04: 74-77	OP2
86.	Introduction and inspiration of training system for doctors without borders foreign aid medical team	Jingmiao Long, etc.<br>School of Public Health, Peking University	China Health Human Resources, 2017,02: 80-83	OP2
87.	Comparative study on the global health strategies of seven OECD countries	Yunping Wang, etc.<br>China National Health Development Research Center, National Health Commission	Chinese Journal of Health Policy, 2014,7(7):9-17.	OP3
88.	International partners analysis in the field of global health: The case from China	Hui Yin, etc. School of Public Health, Peking University	Chinese Journal of Health Policy, 2015,8(1):52-57.	OP2
89.	Ideology and practice of development assistance for health in China	Yunping Wang, etc. China National Health Development Research Center, National Health Commission	Chinese Journal of Health Policy , 2015,8(5):37-43	OP2
90.	Development assistance for health in BRICS countries	Gui Cao, etc. China National Health Development Research Center, National Health Commission	Chinese Journal of Health Policy , 2015,8(5):44-47	OP2
91.	Understand the linkage of environment and health: A case study of China	Wangyun Hu, etc.School of International Studies, Peking University	Chinese Journal of Health Policy, 2015,8(7): 16-21.	OP3
92.	The linkage of global environment and health:	Zhifang Wang, etc.School of International	Chinese Journal of Health Policy,	OP3

No[3].	Title	First Author and His/Her Affiliation	Journal Information[4]	Code of Outputs
	Deepen of the scientific understanding	Studies, Peking University	2015,8(7): 1-8.	
93.	China's participation of global environment and health governance: Opportunities, challenges and policy recommendation	Haibin Zhang, etc.<br>School of International Studies, Peking University	Chinese Journal of Health Policy, 2015,8(7): 21-26.	OP3
94.	The linkage of global environment and health: Policy response and institutional construction	Liang Dong, etc.<br>School of International Studies, Peking University	Chinese Journal of Health Policy, 2015,8(7): 8-16.	OP3
95.	International experience and reference on NGOs and global health	Zhiyuan Hou, etc.<br>School of Public Health, Fudan University	Chinese Journal of Health Policy, 2016,9(11):1-4	OP3
96.	Study on the roles of NGOs in global health	Yi Qian, etc.<br>School of Public Health, Fudan University	Chinese Journal of Health Policy, 2016,9(11):5-10	OP3
97.	Research on the governance tools of international NGOs participating in global health	Minlu Guo, etc. School of Public Health, Fudan University	Chinese Journal of Health Policy, 2016,9(11):11-17	OP3
98.	Government orientation and cooperation with NGOs in global health sector: Example of WHO, US and UK	Zhiyuan Hou, etc. School of Public Health, Fudan University	Chinese Journal of Health Policy, 2016,9(11):18-23	OP3
99.	Experience and references from typical international foundations in global health project management	Yongyi Wang, etc. School of Public Health, Fudan University	Chinese Journal of Health Policy, 2016,9(11):24-30	OP3
100.	Progress and suggestions on health in the post-2015 development agenda	Hui Yin, etc. School of Public Health, Peking University	Chinese Journal of Health Policy, 2015,8(9): 29-34.	OP3
101.	Analysis on the development strategy of the Chinese Global Health NGOs	Minji Zhang, etc. School of Public Health, Fudan University	Chinese Journal of Health Policy, 2016,9(11):31-39	OP3
102.	Establishment of Post-2015 Development Agenda and health related goals	Guanshen Dou, etc.<br>School of Public Health, Fudan University	Chinese Journal of Health Policy, 2016,9(10):50-55	OP3
103.	Experience and implications in the achievement of health-related millennium development goals in China	Xiaoying Chen, etc.<br>School of Public Health, Fudan University	Chinese Journal of Health Policy, 2016,9(5):72-77	OP1
104.	History, current situation and trend of China's overseas health facilities aid	Yunping Wang, etc. China National Health Development Research Center, National Health Commission	Chinese Journal of Health Policy, 2017,10(8):60-67	OP2
105.	Health human resource development cooperation in China's foreign aid: Situation analysis and	Xiaodan Fan, etc. China National Health Development Research Center, National Health	Chinese Journal of Health Policy, 2017,10(8):68-75	OP2

No[3].	Title	First Author and His/Her Affiliation	Journal Information[4]	Code of Outputs
	improvement strategies	Commission		
106.	Cause analysis of the current situation of drug quality in China	Minna Gu, etc. School of Public Health, Fudan University	Chinese Health Resources，2015,18(4):239-243	OP1
107.	Systematic assessment of the youth sex and reproductive health intervention research in China	Mengqi Chen, etc. School of Public Health, Fudan University	Chinese Journal of School Health，2016,37(08):1239-1243	OP1
108.	Cost effectiveness evaluation on comprehensive control measures carrying out in schistosomiasis endemic areas with regard to different layers of administrative villages stratified by infection situation of human and domestic animals Ⅰ Cost - effectiveness study in inner embankment of marshland and lake regions from 2006 to 2010	Huaming Zhang, etc. Jiangling County Center for Disease Control and Prevention	Chinese Journal of Schistosomiasis Control，2014,26(3):254-259	OP1
109.	The evidences for formulation of schistosomiasis control and elimination criteria：results from a large scale of retrospective investigations	Xiaonong Zhou, etc. National Institute of Parasitic Diseases，China CDC	Chinese Journal of Schistosomiasis Control，2014,26(5):479-481	OP1
110.	Endemic status of schistosomiasis in People's Republic of China in 2013	Zhenglong Lei, etc. Disease Control Bureau，National Health and Family Planning Commission	Chinese Journal of Schistosomiasis Control，2014,26(6):591-597	OP1
111.	Cost effectiveness evaluation on comprehensive control measures carrying out in schistosomiasis endemic areas with regard to different layers of administrative villages stratified by infection situation of human and domestic animals Ⅱ Correlation analysis of costs and inputs with changes of schistosomiasis endemic situation in inner embankment of marshland and lake regions from 2006 to 2013	Hehua Hu, etc. Jiangling County Center for Disease Control and Prevention	Chinese Journal of Schistosomiasis Control，2015,27(1):17-21	OP1
112.	Analysis of endemic changes of schistosomiasis in China from 2002 to 2010	Qiang Wang, etc. National Institute of Parasitic Diseases，China CDC	Chinese Journal of Schistosomiasis Control，2015,27(3):229-234	OP1
113.	Implementation of precision control to achieve the	Xiaonong Zhou.	Chinese Journal of Schistosomiasis	OP1

No[3].	Title	First Author and His/Her Affiliation	Journal Information[4]	Code of Outputs
	goal of schistosomiasis elimination in China	National Institute of Parasitic Diseases，China CDC	Control，2016,28(01):1-4	
114.	Analysis of demands of African students for China-Africa malaria prevention training	Qi Wang, etc.School of Public Health, Peking University	Chinese Journal of Schistosomiasis Control，2016,28(4): 461-464	OP2
115.	China’s contribution to research and development of antiparasitic products-Inspiration from Nobel Prize in Physiology or Medicine 2015	Yangmu Huang etc.<br>School of Public Health, Peking University	Chinese Journal of Schistosomiasis Control，2016,28(4):349-352.	OP1
116.	Study on value of IHA in diagnosis of schistosomiasis japonica	Jie Zhou, etc. Hunan Provincial Institute of Schistosomiasis Control	Chinese Journal of Schistosomiasis Control，2016,28(4):375-380	OP1
117.	Playing the guiding roles of national criteria and precisely eliminating schistosomiasis in P.R.China	Jing Xu, etc. National Institute of Parasitic Diseases，China CDC	Chinese Journal of Schistosomiasis Control，2017,29(1):1-4	OP1
118.	Establishment of response system to emergency parasitic disease affairs in China	Chunli Cao, etc.<br>National Institute of Parasitic Diseases，China CDC	Chinese Journal of Schistosomiasis Control，2017,29(4):397-401	OP1
119.	Effect of short-term global health training on tropical diseases and its related factors	Hongmei Li, etc. National Institute of Parasitic Diseases，China CDC	Chinese Journal of Schistosomiasis Control，2018,30(1):1-4	OP1
120.	Development and role of Regional Network for Asian Schistosomiasis and Other Helminth Zoonoses	LijuanZhang, etc. National Institute of Parasitic Diseases，China CDC	Chinese Journal of Schistosomiasis Control，2018,30(1):5-8	OP3
121.	Health Economic Evaluation of Cancer Screening	Fei Wu, etc. School of Public Health, Fudan University	China Cancer，2016,25(02):81-87	OP1
122.	Analysis on AIDS surveillance among MSM in Taizhou,2010-2013	Qionghai Wu, etc.<br>School of Public Health, Fudan University	Chinese Journal of Disease Control & Prevention，2014,18(12):1140-1142	OP1
123.	Analysis on the performance evaluation of the Global Fund Malaria Programme in China from 2003 to 2013	Qingfeng Zhang,etc. National Institute of Parasitic Diseases，China CDC	Chinese Preventive Medicine，2017,51(5):427-431	OP1
124.	Enlightenment and practice of the medical aid of China's evacuation of nationals from Libya	Bo Li, etc. Jiangsu Province CDC	Chinese Journal of Disaster Medicine，2016,4(8):455-457	OP2
125.	Build together the health road of “One Belt, One Road”	Yunping Wang, China National Health Development Research Center, National Health Commission	Chinese Health	OP3

No[3].	Title	First Author and His/Her Affiliation	Journal Information[4]	Code of Outputs
126.	Analysis of the external environment of health cooperation in China, Vietnam and Laos	Si Zhu, etc. School of Public Health, Fudan University	Fudan International Studies Review	OP2
