# Supplementary material for: China-UK partnership for global health: practices and implications of the Global Health Support Programme 2012–2019
Source: Glob Health Res Policy. 2020 Mar 20;5:13. doi: 10.1186/s41256-020-00134-7 (PMC7083009; doi:10.1186/s41256-020-00134-7)
Supplement: Supplementary file 3 — Additional file 3. International Exchange Activities and Training Activities Supported by GHSP. [file 41256_2020_134_MOESM3_ESM.pdf]

Additional file 3 International Exchange Activities and Training Activities Supported by GHSP

Additional file 3-1 International Exchange Activities

Organizing Agencies	Activites[1]	Topics and Contents	Number
Strategic Oversight Committee (SOC) PMO in charge of logistics	China-UK Global Health Dialogue	Discussion and exchange on concerned global health topics between Chinese and British health sectors	5 times, 67 person times
	China-UK Joint Visit to Africa	Participating a joint British-World Health Organization (WHO) study visit in Ethiopia to explore the promotion of China-UK health cooperation in Africa, especially collaboration with WHO Africa and African CDC (2018)	Once, 3 person times
	SOC Overseas Supervision on Pilot Areas	Overseas supervision on pilot areas in Myanmar for 3 times and Tanzania twice	5 times, 41 person times
	Attending International Conference	Attending China-UK Health Policy Dialogue in UK in 2017, the 4th China-Africa Health Cooperation International Symposium in Bozwana in 2013	3 times, 22 person times
	Mid- and Long-term Secondment	Dispatching personnel to work in Global Fund, OECD and WHO in the South Pacific	7 times, 7 person times
OP201 School of Public Health, Peking University	Attending International Conference and Training	Attending the Sixth Annual CUGH Conference, 5th International Symposium on China-Africa Health Cooperation, Liverpool School of Tropical Diseases International Health Consultation Training Course, etc.	5 times, 12 person times
OP202 National Institute of Parasitic	Dispatching Experts to Participate in International Aid Projects	Carrying out the promotion and training of schistosomiasis rapid diagnosis technology in Zambia, assisting the Philippine Department of Agriculture	4 times, 10 person times

[1] The list doesn’t include: (a) overseas trainings organized directly by PMO such DAH flagship training (in Additional file 3-2) and (b) pilot overseas project implementing work.

Organizing Agencies	Activites[1]	Topics and Contents	Number
Diseases, China CDC		Veterinary Bureau to draft and formulate the Philippine animal schistosomiasis medium-term plan, etc.	
	Attending International Conference	Attending the 37th TDR Joint Coordinating Council, on-site application evaluation of schistosomiasis immunological diagnostic reagents, the first forum of the Western Pacific Region of WHOCC, etc.	14 times, 18 person times
	International Secondment for Young Researchers	Secondment in WHO headquarters, the Antwerp Tropical Disease Research Center in Belgium, the London School of Health and Tropical Medicine, etc.	6 times, 10 person times
OP203 China National Health Development Research Center	Attending International Conference	Attending the ASEAN 10+3 National Health Coverage Network First Steering Committee, South-South Development Cooperation: Opportunities and Challenges of the International Aid System Seminar, APEC Health Working Group 2015 First Meeting, etc.	19 times, 35 person times
	Attending International Training	Attending the Global Health Diplomacy Training in Switzerland	Twice, 3 person times
OP301 School of Public Health, Peking University	Attending International Conference	Attending the 5th CUGH Conference, World Health Summit, China-Harvard-Africa Tripartite Partnership Workshop, etc.	14 times, 23 person times
	Attending Short-term International Academic Training	Attending the Intellectual Property: Diplomacy and Global Health Training Course held by Geneva Institute for Advanced International Relations and Development, 2014 Global Health Diplomacy Advanced Course	Twice, 4 person times
OP302 School of Public Health, Fudan University	Supporting Young Researchers to attend International Conference	Attending international meetings in America and Canada	Twice, 2 person times
	Attending International Conference	Attending the CUGH 2015 Annual Meeting, Global Health Strategy Seminar, Global Health Summit Forum, etc.	7 times, 12 person times

Organizing Agencies	Activites[1]	Topics and Contents	Number
	Attending Short- and Long-term International Training	Attending trainings in Duke University, the University of Maryland, International Agency for Research on Cancer of WHO, etc.	12 times, 12 person times
OP404 China CDC	Holding an International Conference	Holding The Consultation with Partners Supporting Public Health Development Meeting in Nigeria	Once,56 person times
**Total**			**109 times, 349 person times**

Additional file 3-2 Training Activities

Name of the Training	Topics and Contents	Source of Trainees	Number	Organizing agencies
Global Health Certification Training	Theoretic knowledge on global health and typical cases	Health decision-makers at various level of governments and relevant researchers from research agencies	3 times, 96 person times	School of Public Health, Peking University
Training Course on Global Health and Diplomatic Practice	Participation of World Health Assembly and observation of global health policy discuss and negotiation at highest level	Selected participants from Global Health Certification Training	3 times, 18 person times	School of Public Health, Peking University
Advanced Training on Global Health Diplomacy and Development Aid	Global Health Diplomacy, Development Aid and relevant knowledge	Government officials and scientific staff in relevant field	3 times, 120 person times	School of Public Health, Peking University
Training Course on Global Health	Basic concept of global health and relevant methods to carry out global health work and studies	Decision-makers and working staff from colleges and universities, national and provincial administrative departments, research institutes, publishers, NGOs, etc.	3 times, 256 person times	School of Public Health, Fudan University

Name of the Training	Topics and Contents	Source of Trainees	Number	Organizing agencies
Training on Global Health International Consulting Service Capacity	Global health sketch, introduction of international consulting service together with approaches and skills	Experts and researchers engaged in global health work, government officials from global health related departments	Once, 39 person times	Global Health Institute, Wuhan University
Health Development Assistance Flagship Training Course	International development theories and practice, global health initiative and challenge, China's Health Development Assistance (HDA) strategy, UK HDA strategy, UK development project design, implementation and management, etc.	Government officials (cross-sectors), researchers and project management personnel, etc.	Twice, 38 person times	First: China National Health Development Research Center and Institute of Development Studies (IDS)<br>Second: Center for Project Supervision and Management, National Health Commission (PMO) and IDS
Training on of Health Development Assistance Consultant Capacity Building	Global health diplomacy, national HDA theories and practice, consultant capacity building	Researchers (potential consultants)	Once, 30 person times	China National Health Development Research Center
Training Course for Management Staff on Health Development Aid	Health diplomacy theory and practice, international development aid theory and practice, and other health development aid knowledge	management personnel from provincial and municipal health administrative units, CDCs, hospitals, college and universities, etc.	3 times, 116 person times	China National Health Development Research Center
Advanced Training on Foreign Aid for Global Health	Strengthening foreign-aid capacities of provincial level parasites diseases prevention and control agencies	Government officials and management staff (parasitic disease prevention and control)	59 person times	National Institute of Parasitic Diseases, China CDC
Training Course on Global Health-Management of	National diplomacy, global health cooperation, VTTDs prevention and control,	Government officials and management staff (VTTDs prevention and control)	93 person times	National Institute of Parasitic Diseases, China CDC

Name of the Training	Topics and Contents	Source of Trainees	Number	Organizing agencies
Vector Transmitted Tropical Diseases	diagnosis and treatment strategies, international major diseases prevention and control cases studies			
Training Course on Global Health Experts on Tropic Diseases for Foreign Aid Pool	Tropic diseases, foreign-aid, skills and other indications	Technical professionals (Tropic diseases prevention and control)	3 times, 60 person times	National Institute of Parasitic Diseases, China CDC
Training on GHSP Policy Brief	Writing GHSP policy briefs and related practice	The researchers from all the GHSP PIAs	Twice, 60 person times	Center for Project Supervision and Management, National Health Commission (PMO)
Training on Writing Academic Articles on the base of GHSP Research Findings	Writing academic articles for publishing on the international journal	The researchers from the GHSP PIAs	Once,35 person times	Global Health Institute of Wuhan University
**Total**			**27 times, 1020 person times**	
