# Supplementary material for: China-UK partnership for global health: practices and implications of the Global Health Support Programme 2012–2019
Source: Glob Health Res Policy. 2020 Mar 20;5:13. doi: 10.1186/s41256-020-00134-7 (PMC7083009; doi:10.1186/s41256-020-00134-7)
Supplement: Supplementary file 4 — Additional file 4. The Full List of Policy Briefings Produced by the GHSP. [file 41256_2020_134_MOESM4_ESM.pdf]

Additional file 4 The Full List of Policy Briefings Produced by the GHSP

No.[1]	Themes of Policy Briefings	Key Words	Code of Outputs
**I. Maternal, Neonatal, and Child Health（MNCH）**			
1.	From Policy Guidelines to Effective Implementation	MNCH. Health System Strengthening.	OP1
2.	Multiple Measures to Alleviate Financial Burden of Facility-Based Childbirth	MNCH. Health system Strengthening. Health Financing.	OP1
3.	Linking Pregnant Women to Health Facilities for Childbirth: Transforming the Role of Traditional Birth Attendants in Rural China	MNCH. Traditional Birth Attendants. Human Resources for Health (HRH) Capacity development.	OP1
4.	The Impact of China's Poverty Alleviation Policies on Child Nutrition Promotion	MNCH. Nutrition	OP1
5.	Sending specialists to first-level referral centers：an effective way to improve obstetric services in rural areas	MNCH. Human Resources for Health (HRH). Capacity development.	OP1
6.	Top-down Strategies to Increase Coverage of Neonatal Hepatitis B Vaccination	MNCH. Routine immunization. Neonatal Hepatitis B Vaccination	OP1
7.	Fully Implementing Expanded Program on Immunization and Improving People's Health	MNCH. Routine Immunization. Health system strengthening.	OP1
8.	The Improvement of Nutrition for Children in Poverty-stricken Areas of China	MNCH. Nutrition	OP1
9.	Resource-integrated and Multilateral Cooperation System for the Prevention of Mother-to-Child Transmission (PMTCT) on HIV, Syphilis and Hepatitis B	MNCH. PMTCT. Aid effectiveness	OP1
10.	Experience and Exploration on Post-Abortion Care in China	MNCH. Post-abortion care	OP1
**II. Infectious Diseases**			

[1] This list only includes the policy briefings published publicly by the GHSP.

No.[1]	Themes of Policy Briefings	Key Words	Code of Outputs
11.	The Evolution of Strategies on Schistosomiasis Prevention and Control in China	Tropical diseases. Schistosomiasis	OP1
12.	The 1-3-7 Strategy: National Malaria Surveillance and Response Strategy in China	Tropical Diseases. Malaria. Health system strengthening.	OP1
13.	Civil Society Organisation (CSO) and its Role in Malaria Prevention and Control in China-Myanmar Border Areas	Tropical diseases. Malaria. CSO. Cross -border	OP2
14.	Experience of Livestock Schistosomiasis Control in China	Tropical diseases: Schistosomiasis	OP1
15.	Challenges on Malaria Prevention and Control in Border Regions of Yunnan, China	Tropical diseases. Malaria	OP1
16.	China's Responses Strategy to Imported Malaria Cases	Tropical diseases. Malaria. Health System Strengthening.	OP101
17.	The Research and Development of Antimalarials in China: Current Status and Implications	Tropical diseases. Malaria. R&D	OP301
**III. Non-communicable Diseases (NCD)**			
18.	Obstacles and Suggestions on Tobacco Control in China	NCD. Tobacco control. China	OP302
19.	Evolvement and Implications of Non-communicable Diseases Control in China	NCD. China	OP302
20.	Policy Recommendations on China's Participation in Global Governance for Non-communicable Diseases	NCD、Global Health Governance	OP302
21.	Implications of Non-communicable Diseases Control Practice in Japan for China	NCD. China. Japan	OP302
22.	"Salt Reduction" in China: Practice and Policy Recommendations	NCD. Salt reduction.	OP302
**IV. Health System Strengthening**			
23.	Establishing and Improving a Basic Medical Insurance Financing System in Rural China	Health system strengthening Health Financing. Health Insurance	OP1
24.	The Evolution of Health System Governance in China	Health system strengthening. Governance	OP1
25.	Financing strategies to promote basic public health service equalization in China	Health system strengthening. Health financing.	OP1
26.	Development of village doctors in China financial compensation and health	Health system strengthening. HRH	OP1

No.[1]	Themes of Policy Briefings	Key Words	Code of Outputs
	system support		
27.	Expanding health insurance coverage for informal sector: What can Tanzania learn from China's experience?	Health system strengthening. Health financing. Health insurance	OP1
28.	What can Tanzania learn from the Chinese experience of Community Health Workers to address the challenge of Human Resource for Health in rural areas?	Health system strengthening. HRH. Community Health Workers. China. Tanzania	OP1
**V Development Assistance for Health / Health Cooperation**			
29.	Different Management Structure of Development Aid	Aid effectiveness. Health system strengthening. Governance	OP2
30.	Reflection of the International Aid Transparency Initiative for Development Assistance for Health effectiveness improvement	Aid effectiveness. Transparency	OP2
31.	China should Actively Engage in Development Aid for Health (DAH) Coordination Mechanism	Aid effectiveness. China	OP2
32.	China's Healthcare Development Cooperation with Africa on Tropical Diseases	Aid effectiveness. Tropical diseases.	OP2
33.	Cross-border AIDS Prevention and Control in Dehong Prefecture, Yunnan Province, China	Aid effectiveness. HIV and AIDS. Cross border	OP3
34.	Analysis of Two Large-Scale International Health Assistance Programmes for China	Aid effectiveness, International Health Assistance Programmes	OP2
35.	China and the "Regional Network for Asian Schistosomiasis (RNAS)"	Aid effectiveness. Tropical diseases, Schistosomiasis	OP2
36.	Practice and Reflections of Jiangsu Medical Team in Malta	Aid effectiveness	OP2
37.	Effectiveness of China's Development Assistance for Health to Uganda	Aid effectiveness. Health system strengthening. China. Uganda	OP2
38.	China Health Aid Complete Projects: Transforming from Infrastructure Construction to Integrated Aid on Functions	Aid effectiveness. China	OP2
39.	China-Laos Cross-border Medical Service Cooperation: Current Status and Implications	Aid effectiveness. Cross-border	OP2

No.[1]	Themes of Policy Briefings	Key Words	Code of Outputs
40.	Taking Mongolian Medicine as a Breakthrough to Promoting China-Mongolia Healthcare Cooperation	Health development cooperation strategy. Traditional medicine	OP2
41.	Health cooperation needs and strategy among China and Viet Nam	Health development cooperation strategy	OP2
42.	Promoting Trade and Cooperation on Traditional Chinese Medicine between China and the Philippines: Challenges, Opportunities and Countermeasures	Health development cooperation strategy. Traditional medicine. Trade	OP2
43.	Strengthening China-Cambodia Health Cooperation with the Focus on Infectious Diseases Prevention and Control	Health development cooperation strategy. Infectious diseases	OP2
44.	Starting the China-Mongolia Soft cooperation in public health by initiating bilateral echinococcosis control	Health development cooperation strategy. NZD	OP2
45.	Promoting Traditional Chinese Medicine in Nepal: How to Break through the Bottleneck?	Health development cooperation strategy. Traditional medicine	OP2
46.	Promote Safe Motherhood in Rural Ethiopia - Findings from Collaborative Efforts on Maternal and Reproductive Health	Health development cooperation practice, NMCH	OP4
**Ⅵ. Health Security- AMR**			
47.	China Should Take More Initiatives in the Global Governance of Antibiotic Resistance	AMR. Global governance. China	OP3
48.	China's Strategies Responding to Artemisinin Resistance in the Lancang-Mekong Sub-region	AM.R. Malaria	OP3
