# Supplementary material for: China-UK partnership for global health: practices and implications of the Global Health Support Programme 2012–2019
Source: Glob Health Res Policy. 2020 Mar 20;5:13. doi: 10.1186/s41256-020-00134-7 (PMC7083009; doi:10.1186/s41256-020-00134-7)
Supplement: Supplementary file 5 — Additional file 5. Overseas Pilot Projects supported by the GHSP. [file 41256_2020_134_MOESM5_ESM.pdf]

Additional file 5

Overseas Pilot Projects supported by the GHSP

__Case 1: Overseas Pilot Project on Malaria Control in Tanzania__

I. Organization：The National Institute of Parasitic Diseases of China CDC (NIPD)

II. Partners： • Ifakara Health Institute, Tanzania; • National Malaria Control Programme, Tanzania; • National Institute for Medical Research, Tanzania

III. Goals:

General goal: To reduce malaria disease burden by over 30% in comparison with that at the beginning of the project in the pilot areas by implementation of Chinese experiences in combination with WHO-T3 strategy.

Specific objectives:

• To strengthen the community-based interventions by adopting WHO-T3 strategy in the pilot areas;

• To strengthen the capacity building in establishment of entomological and parasitological surveillance response system as well as information track systems;

• To strengthen the cost-effectiveness of implementation of Chinese experiences in combination with WHO-T3 strategy and evaluation for the community-based interventions in the pilot areas;

• To summarize the lessons and experiences from this project and make recommendations for future foreign aid policy of China government.

IV. Duration and Location:

The project duration was from May 2015 to June 2017. Two communities of Rufiji, located in southern part of Tanzania, were selected as the pilot areas.

V. Main Activities:

• Improving the malaria cases management by setting up mobile test stations as well as introducing electronic reporting system in the pilot areas for rapidly reducing malaria burden in the high risk population;

• Sharing Chinese experiences and providing the technical support on-site through Chinese staffs working together with local health staffs at the ground levels;

• Improving the capacity on malaria cases management for the local clinicians, health staffs at health facilities and the volunteers in the pilot areas;

• Evaluating the cost-effectiveness of implementing WHO-T3 strategy integrated with Chinese experiences and community-based interventions through the baseline survey, mid-term assessment and end line survey in the study areas;

• Summarizing the lessons and experiences from this pilot project and making recommendations for foreign aid model of China government and for future strategic planning of malaria control and elimination of Tanzania government.

VI. Achievements:

• A total of 76 rounds of 1, 7-mRCT (malaria reactive community-based testing and

treatment) response were implemented and significantly reduced malaria prevalence by >70% in the intervention wards, beyond and above the benefit of long-lasting insecticide-treated nets;

• A successful joint tripartite project development and implementation were achieved through translating China's ''1-3-7'' model on malaria elimination into 1, 7-mRCT response approach on malaria control for rapidly reducing malaria burden in the areas of moderate and high transmission setting in Africa.

• A platform for policy dialogue and formulation has been developed through sharing experiences, technology and products on malaria control and elimination between technical staff, policymakers and practitioners in China and Africa.

• Lessons learned from the implementation of the 1,7-mRCT response approach with the community-based capacity building and local health system strengthening will shape Chinese aid support, leverage the use of the rich and recent Chinese experiences on malaria control and elimination, and position China to be one of the most important partners to support African countries in accelerating malaria control and elimination efforts.

Case 2: Overseas Pilot Project on Maternal and Child Health in Myanmar and Ethiopia

I. Organization：Global Health Institute, Fudan University (FDGHI)

II. Partners：• Marie Stopes International (MSI), UK; • MSI Myanmar (MSIM); • MSI Ethiopia (MSIE); • Institute for Health Sciences, Kunming Medical University; • China-Ethiopia Friendship Tirunesh-Beijing General Hospital;• China Medical Team in Ethiopia

III. Goals:

•To promote the engagement of China in global health development for increased capacity of Chinese institutions to work in Low-income Countries (LICs), to improve professional competence in conducting research and health intervention projects in LICs, and to improve evidence to support Chinese global health development programming.

• To apply the evidence-based successful experience of China and carry out intervention activities for RMNCH in two pilot areas in Myanmar and Ethiopia, in order to increase utilization of RMNCH services, thus improve the health of women and children.

•To explore experience, lessons and effective models from health development cooperation between China and other developing countries through pilot projects, especially the feasibility of implementing multi-party cooperation project with international non-governmental organizations (NGOs) and training of trainers program in collaboration with Chinese Medical Team and Chinese Ethiopia Friendship Hospitals.

IV. Duration and Location:

The project duration was from Nov. 2015 to June 2017 in one district of Ethiopia and from Oct. 2016 to June 2018 in one district of Myanmar. It has covered around 250,000 and 300,000 people in two countries respectively.

V. Main Activities:

• Improving Chinese agencies' ability to work in low-income countries through personnel dispatch and on-site supervision; strengthening professional capacity through training program and "learning by doing" field practices.

• Applying China's successful experience and approaches of RMNCH services, considering the actual situation of pilot countries, developed the pilot intervention activities, which were tailored to local contexts.

• Increasing awareness and utilization of RMNCH services in these two pilot areas by adopting a series of measures, including strengthening the linkage between communities and health facilities through local community mobilisers, overcoming economic and geographic barriers in service accessibility and improving quality of service.

•Carrying out three rounds of training of trainers program and establishing an advanced Midwifery Training Center in Tirunesh-Beijing General Hospital with training equipment and teaching materials.

VI. Deliverables：

• For the intervention effectiveness, in Ethiopia, the coverage of antenatal care increased from 5% to 47% after the project was implemented, whereas it was 27% in rural areas nationwide. The institutional delivery rate in Gonji Kolela improved from 28% to 55%, while the national data for the same period is less than 21%. In Myanmar, the pilot project significantly improved the institutional delivery in Hlaing Thar Yar Township from 30% to 53% in both urban and rural health facilities. There is also an increasing in four-times of quality antenatal care visits and postnatal care visit by 19% and 10% respectively.

• For the partnership building, Chinese universities piloted this project in collaboration with international NGOs and local government in Ethiopia and Myanmar, Chinese Medical Team and China-Ethiopia Friendship Hospital. An advanced midwifery training center was established in Ethiopia and 13 Ethiopian trainees were trained by Fudan University.

• For the Chinese team member capacity strengthening, 46 Chinese team members worked in Ethiopia and Myanmar with a total of 885 person-days. 17 training workshops, 7 joint lectures and 5 individual counseling sessions were organized during project period and a total of 320 person-times were trained.
